# Российские критерии приемлемости назначения менопаузальной гормональной терапии пациенткам с сердечно-сосудистыми и метаболическими заболеваниями. Согласительный документ РКО, РОАГ, РАЭ, ЕАТ, АФР, РНМОТ, РАГГ

**DOI:** 10.14341/probl13694

**Published:** 2026-01-18

**Authors:** Е. В. Шляхто, И. И. Дедов, В. Н. Серов, Г. Т. Сухих, Г. П. Арутюнов, И. А. Сучков, О. М. Драпкина, О. Н. Ткачева, Я. А. Орлова, И. И. Баранов, Е. Н. Андреева, С. В. Юренева, М. И. Ярмолинская, А. А. Сметник, С. В. Виллевальде, Н. А. Козиолова, И. В. Сергиенко, И. С. Явелов, О. Б. Иртюга, О. П. Григорян, Е. Н. Дудинская, И. А. Золотухин, Е. А. Илюхин

**Keywords:** менопаузальная гормональная терапия, сердечно-сосудистые заболевания, метаболические заболевания, сахарный диабет, венозные тромбоэмболические осложнения, menopausal hormone therapy, cardiovascular diseases, metabolic diseases, diabetes, venous thromboembolic complications

## Abstract

Климактерические симптомы могут нарушать ход жизни женщин на пике карьеры и семейной жизни. В настоящее время самым эффективным методом лечения этих проявлений является менопаузальная гормональная терапия (МГТ). Наличие сердечно-сосудистых и метаболических заболеваний не исключает возможность назначения МГТ с целью купирования климактерических симптомов и улучшения качества жизни. Однако нередко препятствием для использования этого вида гормональной терапии являются опасения врачей, которые боятся принести пациенткам больше вреда, чем пользы. Осторожность особенно важна, когда речь идет о женщинах, страдающих сопутствующими заболеваниями. Более того, следует признать, что качественных исследований относительно безопасности МГТ при основных хронических неинфекционных заболеваниях и часто встречаемых коморбидных состояниях недостаточно. В представленном согласительном документе проведен анализ всех доступных в настоящее время данных, полученных в ходе клинических исследований различного дизайна, и создан свод критериев приемлемости назначения МГТ женщинам с сопутствующими сердечно-сосудистыми и метаболическими заболеваниями. Опираясь на представленный документ, врачи различных специальностей, консультирующие женщин в климактерии, получат доступный алгоритм, позволяющий избегать потенциально опасных ситуаций и обоснованно назначать МГТ в клинической практике.

## Abstract (en)

Menopausal symptoms can disrupt the lives of women at the peak of their careers and family life. Currently, the most effective treatment for these symptoms is menopausal hormone therapy (MHT). The presence of cardiovascular and metabolic diseases does not preclude the use of MHT to relieve menopausal symptoms and improve quality of life. However, physicians’ concerns about causing more harm than good often hinder the use of this type of hormone therapy. Caution is especially important when it comes to women with comorbidities. Moreover, it should be acknowledged that high-quality studies on the safety of MHT for major chronic noncommunicable diseases and common comorbid conditions are insufficient. This consensus document analyzes all currently available data from clinical trials of various designs and develops a set of eligibility criteria for prescribing MHT to women with comorbid cardiovascular and metabolic diseases. Based on this document, physicians of various specialties who consult with women in menopause will receive an accessible algorithm that will allow them to avoid potentially dangerous situations and appropriately prescribe HRT in clinical practice.

## СОГЛАСИТЕЛЬНЫЙ ДОКУМЕНТ

Со-председатели:Шляхто Е.В., Дедов И.И., Серов В.Н., Сухих Г.Т., Арутюнов Г.П., Сучков И.А., Драпкина О.М., Ткачева О.Н.

Ответственный секретарь рабочей группы: Орлова Я.А.

Рабочая группа:Баранов И.И., Андреева Е.Н., Юренева С.В., Ярмолинская М.И., Сметник А.А., Виллевальде С.В., Козиолова Н.А., Сергиенко И.В., Явелов И.С., Игрюга О.Б., Григорян О.Р., Дудинская Е.Н., Золотухин И.А., Илюхин Е.А.

Эксперты:Абашова Е.И., Аганезова Н.В., Алиева А.С., Артымук Н.В., Арутюнов А.Г., Бабенко А.Ю., Балан В.Е., Баранова Е.И., Беженарь В.Ф., Бобров С.А., Васюкова О.В., Габидуллина Р.И., Глезер М.Г., Григорьева Н.Ю., Губарева И. В., Густоварова Т.А., Дженина О.В., Доброхотова Ю.Э., Дубровина С.О., Енькова Е.В., Ермакова Е.И., Зырянов С.К., Иловайская И.А., Карахалис Л.Ю., Карева Е.Н., Каткова Н.Ю., Кирсанова Т.В., Коренная В.В., Кузнецова Т.Ю., Кулешов В.М., Макаренко Т.А., Мальцева Л.И., Мальчикова С.В., Мельниченко Г.А., Мингалева Н.В., Недогода С.В., Никулина С.Ю., Обоскалова Т.А., Петрова М.М., Плисюк А.Г., Подзолков В.И., Подзолкова Н.М., Протасова А.Э., Савельева И.В., Сандакова Е.А., Сахаутдинова И.В., Селихова М.С., Соколова Т.М., Сотникова Л.С., Спиридонова Н.В., Табеева Г.И., Тапильская Н.И., Тарловская Е.И., Фомин И.В., Хамошина М.Б., Чесникова А.И., Чумакова Г.А., Шапошник И.И., Шестакова Е.А., Шестакова М.В., Шереметьева Е.В., Ших Е.В.

## ВВЕДЕНИЕ

Распоряжением Правительства РФ от 29.12. 2022 г. № 4356-р утверждена Национальная стратегия действий в интересах женщин на 2023–2030 гг. Одной из важных задач государственной политики становится сохранение здоровья женщин всех возрастов, улучшение качества жизни и увеличение периода активного долголетия [1]. Для реализации этой стратегии в здравоохранении крайне важен междисциплинарный подход. Терапевтам совместно с акушерами-гинекологами необходимо выявлять женщин, вступивших в период менопаузального перехода, для своевременного оказания им необходимой помощи.

Климактерические симптомы могут нарушать ход жизни женщин на пике карьеры и семейной жизни: 75% женщин в возрасте 45–55 лет предъявляют жалобы на приливы; в 28,5% случаев это приливы средней или тяжелой степени выраженности; продолжительность симптомов может составлять 3–15 лет [2].

В настоящее время самым эффективным методом лечения этих проявлений служит менопаузальная гормональная терапия (МГТ). Клинические рекомендации, обновленные РОАГ в 2025 г. «Менопауза и климактерическое состояние у женщины» служат основополагающим документом для врачей всех специальностей, определяющим стратегию ведения пациенток [3][4].

Наличие сердечно-сосудистых (ССЗ) и метаболических заболеваний не исключает возможность назначения МГТ с целью купирования климактерических симптомов и улучшения качества жизни. Однако нередко препятствием для использования этого вида гормональной терапии являются опасения врачей, боящихся принести пациенткам больше вреда, чем пользы.

Осторожность особенно важна, когда речь идет о женщинах, страдающих сопутствующими заболеваниями. Более того, следует признать, что качественных исследований по оценке безопасности МГТ при основных хронических неинфекционных заболеваниях и часто встречаемых коморбидных состояниях недостаточно.

Таким образом, цель согласительного документа: анализ всех доступных в настоящее время данных, полученных в ходе клинических исследований различного дизайна, и создание свода критериев приемлемости назначения МГТ женщинам с сопутствующими ССЗ и метаболическими заболеваниями.

Опираясь на представленный документ, врачи различных специальностей, консультирующие женщин в климактерии, получат доступный алгоритм, позволяющий избегать потенциально опасных ситуаций и обоснованно назначать МГТ в клинической практике.

## РАЗДЕЛ 1. ОСНОВНЫЕ ОПРЕДЕЛЕНИЯ, СИМПТОМЫ И КЛАССИФИКАЦИЯ МЕНОПАУЗЫ

Менструальный цикл — один из важнейших показателей здоровья женщины, и его регулярность может меняться в зависимости от стадии репродуктивного старения.

Рабочая группа по изучению стадий старения репродуктивной системы женщин (Stages of Reproductive Aging Workshop — STRAW) [5] выделяет три стадии репродуктивного старения: репродуктивная стадия, менопаузальный переход и постменопауза. Классификация этапов старения репродуктивной системы женщин STRAW+10 представлена на рис. 1.

**Figure:**
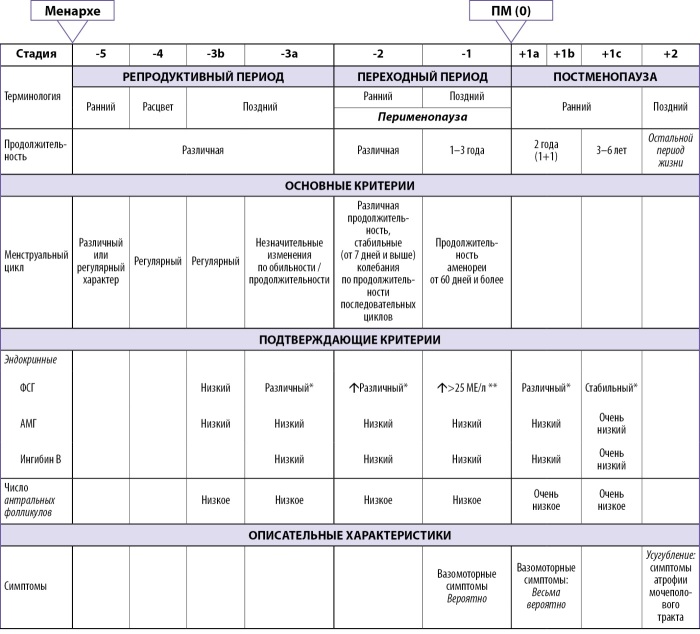
Рисунок 1. Классификация этапов старения репродуктивной системы женщин (STRAW+10). ФСГ — фолликулостимулирующий гормон; АМГ — антимюллеров гормон.

Менопаузальный переход характеризуется нарушением регулярности менструальных циклов, которые служат отражением вариабельности гормональной секреции и овуляторной функции.

Менопауза — стойкое прекращение менструаций, это последняя самостоятельная менструация, обусловленная возрастным снижением гормональной активности и «выключением» репродуктивной функции яичников. Дата наступления менопаузы оценивается ретроспективно: спустя 12 мес отсутствия менструации [6][7].

Перименопауза включает период менопаузального перехода + 1 год после последней менструации.

Перименопауза начинается с нарушения регулярности менструального цикла («фаза менопаузального перехода») и длится до 1 года после полного прекращения менструаций. Эта фаза репродуктивного старения может наступать в широком возрастном диапазоне (от 42 до 58 лет) и длиться 4–8 лет [8].

Постменопауза — период жизни после последней менструации.

Климактерический синдром — комплекс вегетативно-сосудистых, психических и обменно-эндокринных нарушений, возникающих у женщин на фоне угасания (или резкой потери) гормональной функции яичников и общего старения организма [9].

Средний возраст наступления менопаузы во всем мире составляет 48,8 года (95% доверительный интервал — ДИ 48,3–49,2 года) со значительными колебаниями этого показателя в зависимости от географического региона проживания женщин [10], в Российской Федерации он находится в диапазоне от 49 до 51 года [9]. Распространенность климактерических симптомов вариативна и зависит от ряда обстоятельств.

Вазомоторные симптомы чаще возникают в позднем периоде менопаузального перехода и особенно выражены в перименопаузе и первые годы постменопаузы [11][12]. Вазомоторными симптомами страдают почти 80% женщин в перименопаузе [13]. Нарушения сна встречаются у 39—47% женщин в перименопаузе и у 35—60% женщин в постменопаузе [14]. Приливы умеренной или тяжелой степени являются дополнительным, специфическим для лиц женского пола, маркером риска развития метаболических и ССЗ. Наиболее типичная жалоба пациенток с нарушениями сна — частые пробуждения (фрагментация сна). Другими проявлениями служат бессонница, трудности засыпания и ранние пробуждения. Нередко нарушения сна сочетаются с вазомоторными симптомами, а также с повышенной тревожностью, лабильностью настроения.

Среди лиц в возрасте 50 лет и старше в Российской Федерации остеопороз выявляется у 34% женщин, а частота развития остеопении составляет 43% [15].

Вазомоторные симптомы и другие проявления климактерического синдрома не только ухудшают качество жизни женщин и ограничивают их функциональные возможности, но и ассоциированы с повышением риска развития ишемической болезни сердца (ИБС) в 1,34 раза, риска любых ССЗ — в 1,48 раза [16].

У 15% женщин в перименопаузе и почти 80% женщин в постменопаузе отмечаются симптомы генитоуринарного менопаузального синдрома (ГУМС) или вульвовагинальной атрофии (ВВА) [17]. Сухость влагалища, зуд и диспареуния (болезненность при половом акте) являются симптомами, связанными с ВВА.У 41% женщин в возрасте 50–79 лет есть хотя бы один из симптомов ВВА. Распространенность нарушений мочеиспускания (внезапное и непреодолимое желание помочиться, которое невозможно отсрочить, недержание мочи) у женщин зависит от длительности постменопаузы и увеличивается с 15,5% при постменопаузе до 5 лет и до 41,4% при длительности постменопаузы более 20 лет [17].

## Классификация менопаузы

По времени наступления выделяют:

– преждевременную менопаузу или преждевременную недостаточность яичников (до 40 лет);

– раннюю (40–44 лет);

– своевременную (45–55 лет) и

– позднюю (старше 55 лет).

По причине наступления выделяют естественную и ятрогенную (в том числе хирургическую менопаузу).

При преждевременной недостаточности яичников (ПНЯ) после гистерэктомии, на фоне приема комбинированных пероральных контрацептивов, внутриматочной системы, содержащей 52 мг левоноргестрела микронизированного (ЛНГ-ВМС) критерии STRAW+10 неприменимы.

## РАЗДЕЛ 2. ПОКАЗАНИЯ И ПРОТИВОПОКАЗАНИЯ К МГТ

Показания и противопоказания к назначению МГТ определяются актуальными клиническими рекомендациями и инструкциями к конкретным препаратам.

## Показания к назначению МГТ [4]:

– лечение вазомоторных симптомов умеренной и тяжелой степени, существенно снижающих качество жизни;

– лечение симптомов ГУМС, нарушение половой функции;

– профилактика постменопаузального остеопороза;

– восполнение дефицита эстрогенов при ПНЯ и ранней менопаузе, при двусторонней овариэктомии.

## Противопоказания к назначению МГТ [4]:

– кровотечение из половых путей неясного генеза;

– рак молочной железы — РМЖ (диагностированный, подозреваемый или в анамнезе);

– диагностированные или подозреваемые эстрогензависимые злокачественные новообразования (эндометрия, яичников, матки);

– острые и хронические заболевания печени в настоящее время или в анамнезе (до нормализации функциональных проб печени), в том числе злокачественные опухоли печени;

– тромбозы (артериальные и венозные) и эмболии в настоящее время.

– инфаркт миокарда (ИМ);

– ишемические или геморрагические цереброваскулярные нарушения.

– миома матки с субмукозным расположением узла;

– полип эндометрия;

– аллергия к компонентам МГТ;

– кожная порфирия (для эстрогенного компонента);

– прогестагензависимые новообразования (например, менингиома — для гестагенов).

## РАЗДЕЛ 3. ВИДЫ МГТ И ОСНОВНЫЕ ПРИНЦИПЫ ЕЕ НАЗНАЧЕНИЯ

## Монотерапия гестагенами

Женщинам в периоде менопаузального перехода с целью профилактики гиперпластических процессов в эндометрии и регуляции менструального цикла показано применение гестагенов в циклическом режиме на срок не менее 10–14 дней.

В табл. 1 представлены зарегистрированные на территории Российской Федерации препараты для монотерапии гестагенами c целью регуляции менструального цикла и профилактики гиперпластических процессов в эндометрии.

**Table:** Таблица 1. Зарегистрированные в Российской Федерации лекарственные препараты для монотерапии гестагенами

Монотерапия гестагенами
Микронизированный прогестерон 200—300 мгДидрогестерон 10—20 мгПрогестерон и его производные назначаются на срок от 12 до 14 дней

Системная МГТ

Системная МГТ — наиболее эффективный метод лечения вазомоторных симптомов и других климактерических проявлений. Большинство лекарственных препаратов МГТ одобрены для профилактики постменопаузального остеопороза, за исключением ультранизкодозированных форм.

В табл. 2 представлены зарегистрированные на территории Российской Федерации препараты для системной МГТ.

**Table:** Таблица 2. Зарегистрированные в Российской Федерации лекарственные препараты и их комбинации для системной МГТ МГТ — менопаузальная гормональная терапия.

Комбинированная терапия эстроген/гестаген в циклическом режиме (в перименопаузе)
Фиксированные комбинации (эстроген/гестаген)
Эстрадиол/дидрогестерон (1 мг/10 мг; 2 мг/10 мг)
Эстрадиола валерат (2 мг)/левоноргестрел (150 мкг)
Эстрадиола валерат (2 мг)/норгестрел (500 мкг)
Эстрадиола валерат (2 мг)/ципротерона ацетат (1 мг)
Свободные комбинации двух препаратов (эстроген/гестаген)
Эстрадиола валерат 2 мг	Микронизированный прогестерон 200–300 мг 12–14 дней
Дидрогестерон 10–20 мг 14 дней
Эстрадиола гемигидрат гель трансдермальный 0,6 мг/г	Микронизированный прогестерон 200–300 мг 12–14 дней
Дидрогестерон 10–20 мг 14 дней
Эстрадиола гемигидрат гель трансдермальный 0,1% — 0,5 г, 1,0 г, 1,5 г	Микронизированный прогестерон 200–300 мг 12–14 дней
Дидрогестерон 10–20 мг 14 дней
Эстрадиола гемигидрат спрей трансдермальный 1,58 мг/доза (эквивалент 1,53 мг эстрадиола)	Микронизированный прогестерон 200–300 мг 12–14 дней
Дидрогестерон 10–20 мг 14 дней
Монофазная комбинированная терапия эстроген/гестаген в непрерывном режиме (в постменопаузе)
Фиксированные комбинации
Эстрадиол/дидрогестерон (0,5 мг/2,5 мг; 1 мг/5 мг)
Эстрадиол/дроспиренон (0,5 мг/0,25 мг, 1 мг/2 мг)
Свободные комбинации двух препаратов (эстроген/гестаген)
Эстрадиола валерат 2 мг	Микронизированный прогестерон 100–200 мг ежедневно
Прогестерон гель вагинальный 8% 90 мг/доза 2 раза в неделю
Дидрогестерон 10 мг/сут
Внутриматочная система, содержащая 52 мг левоноргестрела микронизированного (ЛНГ-ВМС)
Эстрадиола гемигидрат гель трансдермальный 0,6 мг/г	Микронизированный прогестерон 100–200 мг ежедневно
Прогестерон гель вагинальный 8% 90 мг/доза 2 раза в неделю
Дидрогестерон 10 мг/сут
Внутриматочная система, содержащая 52 мг левоноргестрела микронизированного (ЛНГ-ВМС)
Эстрадиола гемигидрат гель трансдермальный 0,1% — 0,5 г, 1,0 г, 1,5 г	Микронизированный прогестерон 100–200 мг ежедневно
Прогестерон гель вагинальный 8% 90 мг/доза 2 раза в неделю
Дидрогестерон 10 мг/сут
Внутриматочная система, содержащая 52 мг левоноргестрела микронизированного (ЛНГ-ВМС)
Эстрадиола гемигидрат спрей трансдермальный 1,58 мг/доза (эквивалент 1,53 мг эстрадиола)	Микронизированный прогестерон 100–200 мг ежедневно
Прогестерон гель вагинальный 8% 90 мг/доза 2 раза в неделю
Дидрогестерон 10 мг/сут
Внутриматочная система, содержащая 52 мг левоноргестрела микронизированного (ЛНГ-ВМС)
Прочие эстрогены
Тиболон 2,5 мг
Монотерапия эстрогенами (для женщин после гистерэктомии)
Эстрадиола валерат 2 мг
Эстрадиола гемигидрат гель трансдермальный 0,6 мг/г
Эстрадиола гемигидрат гель трансдермальный 0,1% — 0,5 г, 1,0 г, 1,5 г
Эстрадиола гемигидрат спрей трансдермальный 1,58 мг/доза (эквивалент 1,53 мг эстрадиола)

Локальная МГТ

Локальная терапия эстрогенами. Локальная терапия эстрогенами (эстриолом) используется у женщин в пери- и постменопаузальный период с жалобами только на симптомы ГУМС: сухость влагалища, диспареунию или дискомфорт при половой жизни, мочевые симптомы (императивные и/или частые позывы на мочеиспускание, дизурия, рекуррентные инфекции мочевыводящих путей), связанные с этим состоянием [18].

При назначении локальных эстрогенов отсутствуют ограничения по возрасту и длительности постменопаузы. При недостаточном действии системной МГТ на симптомы ГУМС возможно дополнительное назначение локальных эстрогенов (ЛЭ) [19].

Эффективность локального эстриола 0,5 мг в лечении симптомов ГУМС/ВВА доказана в ходе рандомизированных контролируемых исследований (РКИ) и систематических обзоров [20].

Длительные наблюдения (6–24 мес) показывают отсутствие влияния ЛЭ на эндометрий, поэтому дополнительное использование прогестагенов не требуется. ЛЭ не повышают риск развития венозных тромбоэмболических осложнений (ВТЭО), РМЖ, ССЗ, гиперплазии и рака эндометрия по данным наблюдательных исследований [21].

В табл. 3 представлены зарегистрированные на территории РФ препараты для локальной МГТ.

**Table:** Таблица 3. Зарегистрированные в Российской Федерации лекарственные препараты для локальной гормональной терапии

Локальные эстрогены	Эстриол (крем вагинальный 1 мг/г, суппозитории вагинальные 0,5 мг)
Эстриол микронизированный 0,2 мг/прогестерон микронизированный 2 мг/лактобактерии (капсулы вагинальные)
Эстриол 50 мкг/г (гель вагинальный)
Эстриол 0,03 мг/лактобактерии (таблетки вагинальные)
Прочие половые гормоны и модуляторы половой системы	Прастерон (суппозитории вагинальные 6,5 мг)

После отмены ЛЭ симптомы ГУМС возобновляются, что обосновывает необходимость длительного применения [22].

Локальная терапия прастероном. Прастерон (дегидроэпиандростерон) — эндогенное вещество стероидной природы, которое метаболизируется в андрогены и эстрогены только в клетках мочеполового тракта при местном применении. Прастерон имеет минимальный уровень абсорбции. При его применении сохраняются уровни эстрогенов в крови, соответствующие постменопаузальным значениям [23–27].

Основные принципы назначения МГТ:

Назначение, коррекция или отмена МГТ, а также динамический контроль за эффективностью и переносимостью лечения находятся в зоне ответственности акушера-гинеколога.

## РАЗДЕЛ 4. МГТ У ПАЦИЕНТОК С ОЖИРЕНИЕМ, МЕТАБОЛИЧЕСКИМИ НАРУШЕНИЯМИ И НАРУШЕНИЯМИ УГЛЕВОДНОГО ОБМЕНА

Согласно имеющимся научным данным период естественного менопаузального перехода сопровождается постепенным формированием дефицита прогестерона; позднее, в период ранней постменопаузы, уровни эстрогенов и прогестерона резко снижаются [28][29].

Эстрогены играют важную роль в биологии жировой ткани: снижают число рецепторов к андрогенам в висцеральной жировой ткани, препятствуют распределению жировой ткани по висцеральному типу, благоприятно действуют на нейроэндокринный контроль пищевого поведения. Эстрадиол обеспечивает гомеостаз, участвует в регуляции утилизации и сохранения энергии; его дефицит постепенно приводит к развитию менопаузального метаболического синдрома (ММС) [30].

Выраженное снижение концентрации эстрогенов, сопровождающееся формированием относительной гиперандрогении у ряда женщин, рассматривается как основной фактор, вызывающий увеличение массы тела и перераспределение жира в организме у женщин в постменопаузе [31].

При этом выработка андрогенов надпочечниками не меняется, а в яичниках сохраняется продукция андрогенов тека-клетками. Вместе с тем вслед за снижением выработки эстрогенов яичниками снижается уровень глобулина, связывающего половые гормоны, что закономерно приводит к увеличению индекса свободных андрогенов. Кроме того, у женщин в постменопаузе увеличивается синтез андрогенов в периферических тканях внутри клетки в соответствии с концепцией интракринологии. Это способствует формированию физиологического сдвига в сторону относительного преобладания андрогенов над эстрогенами в ранней постменопаузе, при этом уровень тестостерона остается в пределах референсных значений [29][32–34].

В крупном исследовании SWAN (Study of Women’s Health Across the Nation) установлено, что изменение соотношения уровня эстрогенов и андрогенов в период менопаузального перехода, а не динамика их абсолютных значений является предиктором формирования ММС у женщин в дальнейшем [35].

Относительная гиперандрогения тесно связана с метаболическими нарушениями, включая резистентность к инсулину. Инсулин стимулирует синтез андрогенов и, кроме того, способен подавлять выработку глобулина, связывающего половые гормоны, в печени, что приводит к повышению уровней как общего, так и свободного тестостерона. Гиперандрогения, в свою очередь, способствует развитию инсулинорезистентности и висцерального ожирения. Сформировавшийся таким образом порочный круг причинно-следственных нарушений с течением времени может усугублять клинические симптомы гиперандрогении и резистентности к инсулину [29][36–41].

Инсулинорезистентность, дислипидемия, артериальная гипертензия (АГ) и абдоминальное ожирение — основные маркеры ММС [42]. По сравнению с репродуктивным периодом, женщины в период перименопаузы и ранней постменопаузы подвержены более высокому риску прогрессирования инсулинорезистентности [43].

Нарушение секреции инсулина и инсулинорезистентность признаны ключевыми патогенетическими механизмами формирования сахарного диабета (СД) 2-го типа. В свою очередь СД 2-го типа и метаболический синдром являются значимыми факторами риска развития атеросклеротических ССЗ и приводят к увеличению смертности от них [29][44–46]. С возрастом риск развития метаболического синдрома увеличивается у женщин в 5 раз. Распространенность ССЗ повышается у женщин с нарушениями углеводного обмена в 5 раз [47].

Снижение уровня эстрогенов у женщин с наступлением менопаузы сопровождается изменением регуляции энергетического гомеостаза, что приводит к избыточному накоплению жировой ткани [48][49]. Ожирение, особенно абдоминальное, тесно ассоциировано с метаболическим синдромом и значительно повышает риск развития кардиометаболических нарушений, что негативно влияет на прогноз и продолжительность жизни женщин [50].

Помимо дефицита эстрогенов относительная гиперандрогения и активация минералокортикоидных рецепторов в постменопаузе способствуют формированию висцерального ожирения, которое первоначально может носить скрытый характер в случае сохранения нормальной массы тела [29][51].

В свою очередь ожирение является основным ФР ряда других хронических заболеваний, включая СД 2-го типа и ССЗ. Женщины с индексом массы тела (ИМТ) ≥24,9 кг/м² имеют более высокий риск развития СД, а риск смерти от ССЗ у них в 4 раза выше, чем у женщин с нормальным ИМТ [29][37–41][52–56].

Ожирение является независимым ФР развития венозных тромбоэмболических осложнений (ВТЭО). В РКИ WHI (Women’s Health Initiative) у женщин с ожирением (ИМТ >30 кг/м²) отмечено трехкратное увеличение риска развития ВТЭО по сравнении с женщинами с нормальным ИМТ даже в группе плацебо [57].

Для практикующего врача важное значение имеет не только лечение пациентов с сформировавшимися метаболическими нарушениями и ССЗ, но и их первичная профилактика. Важным аспектом является снижение риска развития и обратимость ММС при своевременном изменении образа жизни, контроле/снижении массы тела, а также вовремя начатой патогенетически обоснованной терапии.

При ожирении нежелательно назначать препараты, содержащие гестагены с остаточной андрогенной и глюкокортикоидной активностью, предпочтение отдается метаболически нейтральным прогестагенам [58]. После обнаружения связи минералокортикоидных рецепторов с дифференциацией жировой ткани установлена потенциальная роль прогестерона и прогестинов с антиминералокортикоидными свойствами в контроле массы тела и пролиферации жировой ткани [59].

На фоне снижения уровней эстрогенов у некоторых женщин возможно развитие абдоминального ожирения и повышение риска развития метаболических нарушений, сердечно-сосудистых осложнений (ССО), а также относительной гиперандрогении, которая сопровождается появлением поздних акне, алопеции, гирсутизма [4][60].

В случае развития гирсутизма или акне в период постменопаузы необходимо исключить такие патологии, как стромальная гиперплазия яичников, гормонпродуцирующая опухоль яичников или надпочечников, врожденная гиперплазия коры надпочечников, синдром Иценко–Кушинга, образования щитовидной железы [61]. При выраженных признаках гиперандрогении необходимо углубленное исследование для уточнения причины и назначения патогенетической терапии. При подозрении на указанные состояния целесообразно направить пациентку к эндокринологу для дифференциальной диагностики причин гиперандрогенного состояния и назначения патогенетически обоснованной терапии [4][61]. Изолированные акне без гирсутизма (acne tarda) могут быть не связаны с гиперандрогенией.

Расространенность СД 2-го типа в популяции женщин составляет в 40–44 года — 1,2%, в 45–49 лет — 2,4%, в 50–54 года — 4,2%, в 55–59 лет — 9,4% [62]. Своевременное начало применения МГТ может отложить риск развития СД 2-го типа.

По данным метаанализа 107 исследований, МГТ снижает риск развития СД 2-го типа на 30% (отношение рисков — ОР 0,7; 95% ДИ 0,6–0,9), а при имеющемся СД на фоне МГТ происходит снижение уровня глюкозы в крови и индекса инсулинорезистентности (HOMA-IR), а также наблюдается улучшение липидного состава крови и снижение артериального давления (АД) наряду со снижением степени абдоминального ожирения. На фоне монотерапии эстрогенами или комбинированной МГТ у женщин с СД 2-го типа не отмечено увеличения риска смерти от ССЗ [63].

При СД 2-го типа может быть назначен любой вид МГТ в отсутствие противопоказаний. При назначении комбинированной МГТ важно учитывать метаболические эффекты гестагена, входящего в состав комбинированной МГТ: следует остановить выбор на прогестагенах с нейтральным воздействием на метаболические процессы (микронизированный прогестерон, дроспиренон, дидрогестерон) [64].

Благоприятное влияние МГТ на углеводный обмен прекращается при отмене терапии.

Совместимость сахароснижающей терапии с МГТ, заместительной терапии левотироксином натрия (L-T4), тиреостатической и дофаминергической терапией с учетом путей введения отражена в табл. 4 [65].

**Table:** Таблица 4. Совместимость МГТ и других фармакологических групп в эндокринологии 1 — прием данной терапии на фоне МГТ безопасен, противопоказаний не имеет. 2 — прием данной терапии на фоне МГТ в целом безопасен, может потребоваться подбор дозы одного/двух компонентов. * — при сочетании терапии L-T4 и МГТ может потребоваться коррекция дозировки L-T4 в связи с увеличением нагрузки на глобулин, связывающий половые стероиды, в связи с применением МГТ; ** — прием МГТ не влияет на размер микро/макропролактиномы. МГТ — менопаузальная гормональная терапия; ПО Э/Г —пероальные Эстроген/Гестагены ТД Э/Г трансдермальные Эстроген/Гестагены — ПО Э пероральные Эстрагены—ТД Э— трансдермальные Эстрагены. L-T4 — левотироксин натрия.

Группа препаратов	Комбинированная МГТ	Только эстроген-содержащая МГТ	Тиболон	Локальная МГТ (эстриол или прастерон)
ПО Э/Г	ТД Э/Г	ПО Э	ТД Э
Пероральная сахароснижающая терапия	1	1	1	1	1	1
Инсулинотерапия	1	1	1	1	1	1
L-T4*	1	1	1	1	1	1
Тиреостатики	1	1	1	1	1	1
Агонисты дофамина**	2	2	2	2	2	1

## Заболевания ЖКТ, связанные с нарушением всасывания, у пациенток с метаболическим синдромом

Снижение уровней эстрогенов и прогестерона в постменопаузе может быть ассоциировано с прогрессированием заболеваний желудочно-кишечного тракта (ЖКТ) и усугублять риск развития мальабсорбционных состояний, особенно у пациенток с метаболическими нарушениями [66].

Синдром нарушенного всасывания (мальабсорбция) возникает вследствие дисфункции тонкой кишки, поджелудочной железы с нарушением ее внешнесекреторной функции, заболеваний желчного пузыря [67]. При хронических воспалительных заболеваниях ЖКТ нарушаются процессы всасывания и конъюгации эстрогенов, ухудшается моторика ЖКТ, нарушается секреция желчных кислот, усиливается развитие жировой дистрофии печени, повышается риск формирования камней в желчном пузыре [68][69].

При наличии заболеваний ЖКТ, таких как врожденный дефицит лактазы, непереносимость лактозы, глюкозо-галактозная мальабсорбция следует использовать трансдермальные формы эстрогенов [4][70].

Ключевые положения:

– На фоне дефицита прогестерона и эстрогенов в период ранней постменопаузы, при продолжающейся выработке андрогенов в яичниках и надпочечниках происходит формирование физиологического сдвига в сторону относительного преобладания андрогенов в ранней постменопаузе. При этом абсолютные значения уровня тестостерона соответствуют референсным для стадии репродуктивного старения.

– Физиологический сдвиг в сторону относительного преобладания андрогенов в постменопаузе ассоциирован с увеличением последующего риска развития метаболических и ССЗ, СД 2-го типа.

– При выраженных признаках гиперандрогении необходимо направление к эндокринологу для уточнения причины и назначения патогенетической терапии. При наличии клинических признаков гиперандрогении целесообразно добиваться антиандрогенного эффекта.

– Следует учитывать, что эстрогены обладают уникальной кардиопротективной ролью и прогрессирующее снижение их выработки ассоциировано с повышением риска развития ССЗ.

– МГТ оказывает благоприятное влияние на состав тела за счет уменьшения висцерального ожирения.

– В сочетании с применением МГТ у женщин с ожирением рекомендуется проводить образовательные беседы с целью коррекции привычного образа жизни и снижения массы тела.

– У женщин с сохраненной маткой следует остановить выбор на прогестагенах с нейтральным воздействием на метаболические процессы.

Своевременно начатая МГТ может отсрочить развитие СД 2-го типа.

– Пациенткам с СД 2-го типа может быть назначена МГТ. Выбор режима, состава и пути введения МГТ осуществляется на основе персонифицированного подхода.

– МГТ оказывает положительное влияние на гликемический профиль у женщин как без СД, так и с СД 2-го типа.

## РАЗДЕЛ 5. МГТ У ПАЦИЕНТОК С ТРОМБОФИЛИЯМИ, ЗАБОЛЕВАНИЯМИ ВЕН, ВЕНОЗНЫМИ ЭМБОЛИЯМИ

## 5.1. Состав МГТ и риск развития венозных тромбоэмболических осложнений

Считается, что МГТ с использованием в ее составе пероральных эстрогенов повышает риск развития ВТЭО — тромбоза глубоких вен (ТГВ) и тромбоэмболии легочных артерий (ТЭЛА) [71]. Однако этот эффект, отмеченный в РКИ и выполненных на их основе метаанализах, может быть во многом связан с назначением «тромбогенных» препаратов на основе конъюгированных эквинных эстрогенов (КЭЭ) и медроксипрогестерона ацетата (МПА), а также с несвоевременным началом МГТ [72].

Так, по данным ретроспективного анализа баз данных QResearch и CPRD, выполненного с использованием метода случай—контроль, назначение комбинированной МГТ КЭЭ в сочетании с МПА ассоциировалось с наиболее высоким риском развития ВТЭО. Для перорального эстрадиола отмечено достоверное повышение риска развития ВТЭО и этот эффект был дозозависимым. В то же время для комбинации перорального эстрадиола с дидрогестероном риск развития ВТЭО не увеличивался вне зависимости от режима и дозы эстрадиола. Назначение трансдермального эстрадиола не было связано с увеличением риска развития ВТЭО как при монотерапии, так и в составе комбинированной МГТ. Вне зависимости от ИМТ назначение перорального эстрадиола в комбинации с дидрогестероном, трансдермального эстрадиола как в монотерапии, так и в комбинации с гестагеном, не было связано с увеличением риска развития ВТЭО. В когорте женщин, имевших в анамнезе эпизоды ВТЭО и/или получающих терапию антикоагулянтами, отмечено достоверное снижение риска развития ВТЭО при назначении трансдермального эстрадиола в монорежиме, а также отсутствие увеличения риска развития ВТЭО при комбинированном использовании трансдермального эстрадиола с гестагеном и перорального эстрадиола с дидрогестероном [73].

По данным наблюдательных исследований, на фоне применения трансдермального эстрадиола как в низких (<50 мкг/сут), так и в более высоких дозах в монорежиме, а также его сочетания с любым гестагеном в циклическом или непрерывном режимах, риск развития ВТЭО не увеличивался [73–76].

РКИ надлежащего методологического качества или иные клинические исследования по сопоставлению этих подходов пока отсутствуют.

В наблюдательном исследовании EURAS-HRT (более 30 тыс. женщин) был подтвержден долгосрочный профиль безопасности препаратов, содержащих дроспиренон, для МГТ в отношении ВТЭО. Риск развития ВТЭО на фоне МГТ с дроспиреноном был сопоставим, а риск развития клинически значимых артериальных тромбоэмболических осложнений (главным образом острого ИМ и ишемического инсульта) был достоверно ниже, чем при приеме другой МГТ (детальное сопоставление по составу и особенностям другой МГТ не проводилось) [77].

По имеющимся данным, применение трансдермальных эстрогенов не ассоциировано с повышенным риском развития ВТЭО. У женщин с ФР развития ВТЭ использование трансдермальных эстрогенов не повышало риск ВТЭ [78].

Следует отметить, что в ретроспективном исследовании Y. Vinogradova и соавт. (2019) [73] авторы указали на недостаточность данных для оценки современных препаратов для МГТ (эстрадиол с дроспиреноном, эстрадиол с норгестрелом или левоноргестрелом) и их сравнения с трансдермальной терапией. Исследования, показавшие, по мнению авторов, более высокий риск развития ВТЭО при применении пероральных препаратов в сравнении трансдермальными, относятся к периоду до 2012 г. В последнем систематическом обзоре по данной теме M.S. Goldstajn и соавт. (2023) [74] авторы отмечают, что данные, сравнивающие трансдермальный и пероральный способы введения МГТ, ограничены и имеют низкое методологическое качество, что требует проведения дополнительных исследований.

В рекомендациях Североамериканского менопаузального общество (The North American Menopause Society) 2022 г. также отмечается, что на до сих пор неизвестно, связан ли непероральный путь применения препаратов МГТ с более низким риском развития ВТЭО, РМЖ и ССО, при этом выбор прогестагена также может повлиять на риск развития ВТЭО [70].

В целом современная низкодозированная и ультранизкодозированная комбинированная пероральная МГТ с использованием эстрадиола представляется безопасной в отношении ВТЭО и по риску венозных тромбозов сопоставимой с трансдермальной МГТ [70][73]. Однако оценка пользы и риска при назначении МГТ, выбор лекарственного препарата, его состава и пути введения должны проводиться индивидуально, с учетом особенностей клинической картины и наличия ФР развития ВТЭО.

По данным ретроспективного анализа баз данных QResearch и CPRD, выполненного с использованием метода случай–контроль, не было отмечено увеличения риска развития ВТЭО для тиболона [73].

Локальная терапия эстрадиолом в связи с симптомами ГУМС и прастероном (ДГЭА) — по причине симптомов ВВА не приводит к увеличению риска венозных тромбозов и может использоваться у пациенток всех категорий [65][79].

ЛНГ-ВМС, содержащая 52 мг микронизированного левоноргестрела, также может быть использована как компонент МГТ. По данным исследований, применение ЛНГ-ВМС не приводило к повышению риска развития ВТЭО [80][81].

При принятии решения о возможности и составе МГТ следует учитывать, что риск развития ВТЭО нельзя рассматривать отдельно от других рисков развития тромбозов. Так что даже в случаях, когда не исключено некоторое повышение риска развития ВТЭО, этот эффект может нивелироваться снижением частоты артериальных тромбозов и других ССО, что в итоге обеспечит нейтральное или положительное воздействие на смертность [71][81][82].

Ключевые положения:

– Современная трансдермальная, содержащая эстрадиол, как в монорежиме, так и в комбинации с гестагеном, а также низкодозированная и ультранизкодозированная комбинированная пероральная МГТ с использованием эстрадиола безопасна в отношении ВТЭО.

– МГТ обеспечивает положительный баланс между снижением и повышением риска развития различных тромботических осложнений в пользу снижения частоты развития ССО, что обеспечивает нейтральное или положительное воздействие на смертность.

## 5.2. МГТ в различных клинических ситуациях, связанных с тромбозами

Венозные тромбозы

МГТ при остром ТГВ и/или ТЭЛА противопоказана.

Большинство экспертов рекомендуют отказаться от МГТ и у пациенток с ВТЭО в анамнезе [65][81][84]. Есть свидетельства отсутствия увеличения риска рецидива ВТЭО при трансдермальной МГТ на фоне лечения антикоагулянтами, однако данные о безопасности такого подхода после ВТЭО ограничены [76][78].

При тяжелых менопаузальных симптомах помимо локального применения эстрогенов возможно применение минимальной эффективной дозы трансдермального эстрадиола (<50 мкг/сут). Не исключается возможность применения ультранизкодозированной (0,5 мг эстрадиола) пероральной комбинированной МГТ при соответствующей антикоагулянтной терапии [75][76][83][84]. Не исключено также, что современная МГТ достаточно безопасна после планового прекращения использования антикоагулянтов у больных отдельных категорий с низким риском рецидива венозных тромбозов [76].

Имеющиеся данные не позволяют однозначно судить о риске, связанном с МГТ при остром тромбозе поверхностных вен (ТПВ) голени и ТПВ в анамнезе [85]. Решение о возможности применения современной пероральной и трансдермальной МГТ при ТПВ должно приниматься индивидуально, с учетом особенностей клинической ситуации, наличия ФР развития ВТЭО, а также наличия ТПВ в анамнезе как противопоказания к применению в инструкции к конкретному препарату.

В исследованиях по оценке риска развития ТГВ и/или ТЭЛА после перенесенного ТПВ не проводится разделение между тромбозом неварикозных и тромбозом варикозных поверхностных вен (варикотромбофлебитом). Варикотромбофлебит в первую очередь обусловлен наличием варикозного расширения вен, которое может быть устранено задолго до назначения МГТ.

Варикотромбофлебит в анамнезе следует считать ограничением для назначения МГТ при прямом указании на ТПВ в анамнезе как на противопоказание к применению в инструкции к конкретному препарату для МГТ.

Варикозное расширение вен

Наличие варикозного расширения вен не является противопоказанием к МГТ и не должно влиять на принятие решения о назначении МГТ. В настоящее время нет данных, что МГТ увеличивает риск развития тромбоза варикозно измененных вен (варикотромбофлебита). Проведение ультразвукового исследования вен нижних конечностей перед назначением МГТ не требуется.

Тромбофилии

Данных о безопасности МГТ при антифосфолипидном синдроме очень мало [78]. Из-за высокого риска венозных и/или артериальных тромбозов пероральная и трансдермальная МГТ у больных с антифосфолипидным синдромом не рекомендуется. Потенциально ее возможность не исключена у женщин с невысокой активностью заболевания или бессимптомными изменениями отдельных лабораторных показателей, не имеющих дополнительных ФР тромбозов [85].

Данные о безопасности МГТ при бессимптомных тромбофилиях ограничены. В некоторых исследованиях установлен повышенный риск развития ВТЭО при пероральной МГТ на фоне ряда тромбофилий (дефицит протеина С, дефицит протеина S, дефицит антитромбина, лейденская мутация гена фактора V, мутация гена протромбина G20210A, высокий уровень фактора свертывания крови VIII) [76][86]. Однако этого недостаточно для однозначного запрета пероральной МГТ на фоне бессимптомной тромбофилии, требуются дополнительные исследования данного вопроса.

Применение трансдермальных эстрогенов не повышает риск для женщин — носительниц генов протромботических мутаций (полиморфизмов фактора V — лейденская мутация, и гена протромбина G20210A) [76][78][86].

Решение о возможности назначения и составе МГТ следует принимать индивидуально с учетом сведений о наличии ранее выявленной бессимптомной тромбофилии, тяжести менопаузальных симптомов, наличия дополнительных ФР развития ВТЭО, а также указания определенных тромбофилий в перечне противопоказаний в инструкции к конкретному препарату для МГТ [70][76]. Обследование в целях выявления тромбофилий перед началом МГТ не рекомендуется.

Семейный анамнез тромбозов (венозный или артериальный тромбоз у родственников I степени родства в возрасте до 50 лет) указывает на повышенный риск развития ВТЭО, однако не служит основанием для запрета МГТ [17][70]. В рекомендациях NAMS 2022 г. и согласительном документе MHT Eligibility Criteria Group 2022 г. нет ограничений по семейному анамнезу в отношении какой бы то ни было формы МГТ [65][70].

В рекомендациях ASH 2023 г. в вопросе принятия решения о возможности назначения МГТ выделяются тромбофилии высокого риска развития ВТЭО (дефицит протеинов С, S или антитромбина), рассматриваются сценарии необходимости тестирования на указанные тромбофилии и рекомендуется воздержаться от назначения МГТ при их выявлении [87].

Для пациентки, имеющей родственников I или II степени родства с лабораторно подтвержденным дефицитом протеинов С, S или антитромбина, целесообразно проведение анализа на установленную в семье тромбофилию. При подтверждении наличия этих тромбофилий у обследуемой следует воздержаться от назначения эстрогенсодержащих препаратов. При исключении этих тромбофилий противопоказаний к проведению какой бы то ни было МГТ нет.

Для пациентки с семейным анамнезом тромбозов, в отсутствие в личном анамнезе ситуаций повышенного риска развития ВТЭО, связанных с изменением баланса женских половых гормонов (беременность, прием пероральных контрацептивов), целесообразно тестирование на дефицит протеинов С, S и антитромбина для принятия решения о возможности проведения МГТ. При выявлении этих тромбофилий следует воздержаться от назначения эстрогенсодержащих препаратов. При исключении этих тромбофилий противопоказаний к проведению какой бы то ни было МГТ нет. Принятие решения о целесообразности тестирования является прерогативой лечащего врача.

По имеющимся данным, трансдермальная МГТ не увеличивает риск развития ВТЭО у женщин с бессимптомной тромбофилией, однако свидетельства в пользу ее безопасности в этой клинической ситуации ограничены [76][78][86].

Ограничением для применения конкретного препарата является указание на семейный тромботический анамнез и/или наличие определенных тромбофилий как противопоказание к применению в инструкции.

Ключевые положения:

– Хронические заболевания вен, в том числе варикозное расширение вен нижних конечностей, не являются противопоказанием или ограничением для проведения МГТ.

– Абсолютным противопоказанием к проведению пероральной комбинированной МГТ служит острый ТГВ или ТЭЛА.

– Семейный анамнез тромбозов не является противопоказанием к проведению МГТ. Наличие семейного анамнеза тромбозов может быть основанием для проведения тестирования на дефициты протеинов С, S и антитромбина. Принятие решения о целесообразности тестирования является прерогативой лечащего врача.

– Наличие бессимптомной тромбофилии (без тромботического осложнения в личном анамнезе), за исключением дефицита протеинов С, S и антитромбина, не является противопоказанием к проведению МГТ.

## 5.3. МГТ при хирургических вмешательствах и госпитализации с острым нехирургическим заболеванием

В настоящее время нет доказательств пользы от отмены МГТ перед хирургическими вмешательствами или при госпитализации по поводу острого нехирургического заболевания (кроме тех, при которых МГТ противопоказана) [88].

При стратификации риска развития ВТЭО у подобных больных с помощью соответствующих шкал (например, шкала Каприни для оценки риска послеоперационных ВТЭО, шкалы Падуа (Padua) или IMPROVE VTE у госпитализированных нехирургических пациентов) продолжение МГТ рекомендуется рассматривать как дополнительный ФР развития ВТЭО. Тромбопрофилактика проводится в соответствии с рассчитанным риском и при необходимости обеспечивается назначением антикоагулянтов, которые полностью нивелируют протромботический эффект гормональных препаратов. Отмена МГТ при хирургических вмешательствах не требуется.

Ключевые положения:

– При госпитализации по поводу острого нехирургического заболевания или при проведении планового или экстренного хирургического вмешательства отмена МГТ не требуется.

## РАЗДЕЛ 6. МГТ У ПАЦИЕНТОК С АТЕРОСКЛЕРОТИЧЕСКИМИ СЕРДЕЧНО-СОСУДИСТЫМИ ЗАБОЛЕВАНИЯМИ

В 1998 г. исследование HERS (Heart and Estrogen/progestin Replacement Study), первое плацебо-контролируемое РКИ гормональной терапии (ГТ) эстрогенами и прогестином для вторичной профилактики ИБС у женщин в постменопаузе с установленной ИБС, не выявило пользы в отношении развития ССО и общей смертности при использовании ГТ. Результаты этого исследования являются аргументом против начала ГТ для вторичной профилактики ИБС [89].

Более поздний метаанализ 19 РКИ с участием 40 410 женщин в постменопаузе, получавших МГТ, не выявил значительного увеличения смертности от всех причин, от ССЗ или ИМ на фоне МГТ в рамках как первичной, так и вторичной профилактики ССО. Анализ в подгруппах, основанный на сроках начала МГТ, показал следующее [72]:

– у женщин, начавших МГТ в течение 10 лет после менопаузы, была более низкая смертность (ОР 0,70; 95% ДИ 0,52–0,95) и меньшее число ССО (смерть от ССЗ и несмертельного ИМ; ОР 0,52; 95% ДИ 0,29–0,96);

– у женщин, начавших МГТ >10 лет от начала менопаузы, риск развития инсульта повышался без влияния на смертность или другие исходы ССЗ.

Сравнительных исследований по оценке безопасности тех или иных видов МГТ очень немного. В наблюдательном исследовании EURAS-HRT (более 30 тыс. женщин) при приеме МГТ с дроспиреноном риск развития острого ИМ и ишемического инсульта был достоверно ниже, чем при приеме другой МГТ. Однако детального сопоставления по составу и путям введения МГТ не проводилось [77]. В ретроспективном когортном исследовании с участием 268 596 корейских женщин показано увеличение риска развития ССЗ при приеме тиболона; прием эстрогена как отдельно, так и в комбинации с прогестагеном не увеличивал этот риск; дидрогестерон в составе комбинированной МГТ с эстрогеном был ассоциирован с достоверно более низким риском развития ССО [90]. В настоящее время начало МГТ не рекомендовано женщинам с установленным диагнозом ИБС, включая стенокардию [70], а ИМ служит противопоказанием к МГТ. Манифестация ИБС на фоне приема МГТ, как правило, предполагает ее отмену. Хотя авторы упомянутого исследования HERS (Heart and Estrogen/progestin Replacement Study) по его результатам заключают, что, учитывая благоприятную картину ишемических событий после нескольких лет МГТ, женщинам с ИБС, получающим это лечение, можно его продолжить [91]. Метаанализ, включивший 5766 пациенток с имеющимися ССЗ, показал, что абсолютный риск смерти, развития ИМ, стенокардии или реваскуляризации у больных этой категории на фоне МГТ был низким (табл. 5). Таким образом, у пациенток с развившейся в процессе терапии ИБС, настроенных на продолжение МГТ, вопрос о ее отмене должен быть решен индивидуально совместно кардиологом и гинекологом.

**Table:** Таблица 5. Риск развития сердечно-сосудистых осложнений и смерти при гормональной терапии у пациенток в постменопаузе с сердечно-сосудистыми заболеваниями (данные метаанализа рандомизированных контролируемых исследований) ОР — отношение рисков; ДИ — доверительный интервал.

Показатель	ОР	95% ДИ
Смерть от всех причин	1,04	0,87–1,24
Смерть от сердечно-сосудистых заболеваний	1,00	0,78–1,29
Инфаркт миокарда	0,98	0,81–1,18
Стенокардия	0,91	0,74–1,12
Реваскуляризация	0,98	0,63–1,53
Инсульт	1,09	0,89–1,33

У пациенток с инсультом в анамнезе рекомендуется избегать системной МГТ и требуется рассмотреть альтернативное (негормональное) лечение. В РКИ WHI (Women’s Health Initiative) повышенный риск развития ишемического инсульта отмечен как в группе комбинированной МГТ (ОР 1,37; 95% ДИ 1,07–1,76), так и в группе монотерапии эстрогенами (ОР 1,35; 95% ДИ 1,07–1,70), независимо от исходного риска [91][92]. В мета-анализе 4 исследований, включивших 719 участниц без ССЗ, риск развития инсульта повышался на МГТ (ОР 1,32; 95% ДИ 1,12–1,56) по сравнению с плацебо. В метаанализе исследований, выполненных в рамках вторичной профилактики ССЗ (5172 участницы в 5 исследованиях), отмечена тенденция к увеличению риска развития инсульта (см. табл. 6.1) [72]. Неатеросклеротическая/нетромботическая ИБС чаще встречается у женщин, однако в настоящее время нет достаточно данных для стратификации риска, связанным с применением МГТ, по подтипам заболевания. Для женщин 50–59 лет с ИМ в анамнезе без обструктивного поражения коронарных артерий (КА), спонтанной диссекции КА, коронарной микрососудистой дисфункции или коронарного вазоспазма требуется индивидуальный подход к назначению МГТ. Рекомендуется избегать системной МГТ при спонтанной диссекции КА из-за предполагаемой патофизиологической связи с уровнем женских половых гормонов. Эта рекомендация исходит из того, что >90% пациентов со спонтанной диссекцией КА — женщины.

При симптомах ГУМС у женщин с ССЗ может применяться локальная терапия эстриолом [4][21][70]. Необходимо обратить внимание, что в инструкциях к эстрогенам для локального применения содержаться те же противопоказания, что и к эстрогенам для системной МГТ. Это предупреждение основано не на данных научных исследований, а связано с международными требованиями обязательного указания единых противопоказаний для препарата, независимо от путей его введения [83]. Эстриол при локальном применении характеризуется минимальной системной абсорбцией и не метаболизируется в более активные формы эстрогенов (эстрадиол и эстрон), а уровни циркулирующего эстриола, эстрадиола и эстрона сохраняются в пределах нормальных значений для постменопаузы [93][94]. Результаты нескольких крупных обсервационных исследований подтвердили отсутствие повышенного риска неблагоприятных последствий для здоровья, включая ССЗ, ВТЭО и рак при использовании локальной МГТ эстриолом [95][96].

Ключевые положения:

– МГТ не рекомендована пациенткам с ИБС, а также с перенесенным острым нарушением мозгового кровообращения или транзиторной ишемической атакой. Для лечения вазомоторных симптомов у этих пациенток должна применяться негормональная терапия.

– У пациенток с ИБС, развившейся в процессе МГТ, настроенных на ее продолжение, вопрос об отмене МГТ должен быть решен индивидуально в рамках консилиума, включающего кардиолога и гинеколога.

– Отсутствуют ограничения при назначении локальной МГТ для лечения симптомов ГУМС.

## РАЗДЕЛ 7. МГТ У ПАЦИЕНТОК С ФАКТОРАМИ РИСКА РАЗВИТИЯ СЕРДЕЧНО-СОСУДИСТЫХ ОСЛОЖНЕНИЙ

## 7.1. Дислипидемии

Клинические исследования показали, что по сравнению с плацебо или отсутствием лечения МГТ может значительно повысить уровень холестерина липопротеидов высокой плотности (ХС ЛВП), а также снизить уровень общего холестерина (ОХС), холестерина липопротеидов высокой плотности (ХС ЛНП) и липопротеида-а — Лп(а) [97][99]. Следует отметить, что Лп(а) является независимым ФР развития ССЗ и, в частности повторного ишемического инсульта [100][101]. Статинотерапия оказывает слабое влияние на уровень этого проатерогенного липопротеида, о влиянии на него МГТ данные ограничены. В рамках анализа данных 90 тыс. пациенток в первичной профилактике не обнаружено достоверных различий по снижению уровня Лп(а) у пациенток, получавших и не получавших МГТ [102]. Противоречивые данные имеются в отношении действия МГТ на уровень триглицеридов (ТГ). В части исследований имело место достоверное повышение уровня ТГ [103], а в других работах не было обнаружено существенной разницы уровня ТГ между двумя группами, принимающих плацебо и МГТ [97][103–112].

В целом МГТ рассматривается как терапия, связанная с благоприятными изменениями параметров липидного обмена как при кратковременном, так и при длительном применении у женщин в постменопаузе. Однако есть особенности, связанные с дозами препаратов и способом их доставки.

Показано, что пероральная МГТ увеличивает концентрацию ТГ по сравнению с трансдермальной МГТ [99]. Умеренное, но достоверное повышение уровня ТГ даже на фоне терапии фенофибратом и/или полиненасыщенными жирными кислотами может оказать клинически значимое воздействие как на прогрессирование атеросклероза, так и на развитие панкреатита. Таким образом, для женщин с гипертриглицеридемией более безопасным выбором являются трансдермальная или низкодозированная МГТ либо тиболон.

В то же время пероральная МГТ связана с положительным влиянием на уровень ХС ЛНП, а концентрация именно этого проатерогенного фактора в наибольшей степени влияет на развитие атеросклероза и дестабилизацию атеросклеротических бляшек.

Вопрос о том, может ли МГТ в низких дозах оказывать такое же влияние на липидный состав крови, как и стандартные дозы МГТ, все еще остается неясным. Одно исследование показало, что по сравнению со стандартными дозами низкие дозы МГТ были связаны с более высокими уровнями ОХС и ХС ЛНП, более низким уровнем ТГ [113]. Другие исследования показали аналогичное преимущество в отношении ТГ в группе низких доз эстрогенов в составе МГТ, но не выявили существенных различий по уровням ОХС и ХС ЛНП между двумя группами (высоких и низких доз).

Кроме того, обнаружено, что низкие дозы эстрадиола в составе МГТ могут снижать уровень ХС ЛВП. Эпидемиологически низкий уровень ХС ЛВП в плазме был связан с повышенным риском развития ишемических ССЗ [114]. В совокупности преимущество низких доз МГТ и трансдермального пути введения эстрадиола в отношении липидного состава крови, возможно, ограничивается только уровнем ТГ.

Существуют противоречивые данные о влияния тиболона на липидный составкрови. Метаанализ, проведенный в 2021 г., показал, что тиболон снижает уровни ОХС, ХС ЛВП и ТГ. Концентрации ХС ЛНП значительно снижаются, если прием тиболона длится ≥26 нед [115]. В отношении влияния на Лп(а) различий между обычной МГТ и тиболоном не наблюдалось [116].

Имеются данные о повышенном риске развития ИБС у женщин, получавших комбинированную эстроген-гестагенную терапию, в отличие от женщин, получавших монотерапию эстрогенами [117]. К сожалению, ни в одном крупном РКИ липидный состав крови не оценивался в зависимости от типа используемого прогестагена. Одно из обсервационных исследований показало, что добавление прогестагенов ослабляет благоприятное влияние эстрогена на липидный состав крови [118], а метаанализ, проведенный в 2017 г., выявил отсутствие существенной разницы в снижении концентрации Лп(a) [115].

Хотя результаты ряда исследований продемонстрировали положительное влияние МГТ на липидный состав крови, необходимо подчеркнуть, что МГТ не рекомендуется для терапии дислипидемии и снижения риска развития ССЗ [119].

Ключевые положения:

– МГТ положительно влияет на липидный состав крови у женщин в пери- и постменопаузе.

– МГТ не рекомендуется в качестве терапии дислипидемии, поскольку изменения липидного состава крови на фоне МГТ минимальны и не сопоставимы с эффектами гиполипидемических препаратов.

– Пероральная МГТ может быть предпочтительным выбором у женщин с повышенным уровнем ХС ЛНП.

– Для женщин с гипертриглицеридемией более безопасным выбором являются трансдермальная, ультранизкодозированная МГТ или тиболон.

## 7.2. Артериальная гипертензия

Специфичные для женщин ФР развития АГ и ССЗ в более позднем возрасте включают время наступления менархе, указания в анамнезе на нарушения менструального цикла и репродуктивной функции, миому матки, синдром поликистозных яичников, эндометриоз, неблагоприятные исходы беременности, ПНЯ и менопаузу. Повышенный риск в течение репродуктивного периода жизни может способствовать более значительному увеличению риска развития ССЗ в пери- и постменопаузе [120–124].

При АГ, как и при других заболеваниях, выделяют половые и гендерные различия, которые оказывают влияние на эпидемиологию, патофизиологию и клиническое ведение.

В 2019 г. стандартизированная по возрасту распространенность АГ (систолическое АД ≥140 мм рт.ст. и/или диастолическое АД ≥90 мм рт.ст., или прием антигипертензивной терапии) во всем мире у женщин составила 32% [125]. При этом в Восточной Европе распространенность АГ у женщин в возрасте 30–79 лет колебалась между 34 и 46% [125]. Распространенность АГ увеличивается с возрастом [126], но имеет более выраженную тенденцию к снижению до наступления менопаузы у женщин по сравнению с таковой у мужчин того же возраста, с заметным повышением у женщин после наступления менопаузы [14]. После 65 лет распространенность АГ у женщин выше, чем у мужчин [125–127].

Траектории изменения в течение жизни у мужчин и женщин объясняются различиями механизмов регуляции АД, сочетанием половых и гендерных факторов [125][126]. У женщин до наступления менопаузы эстрогены способствуют снижению АД в контексте их общего вазопротекторного действия. Защита опосредована различными механизмами, в том числе эндотелиальной вазодилатацией за счет усиления пути выработки оксида азота и ингибирования активности симпатической нервной системы и ренин-ангиотензиновой системы. Более того, эстрогены уменьшают выработку эндотелина, окислительный стресс и воспаление [124]. Прекращение функции яичников в результате естественного старения или хирургической менопаузы связано с повышенным бременем ФР кардиометаболических нарушений, включая увеличение массы тела, уровней глюкозы и ОХС в плазме крови, АД, что приводит к повышению риска развития ССЗ [123][124][128][129]. После менопаузы заметное снижение уровня эстрогенов частично объясняет, почему уровень АД и риск развития АГ увеличиваются [124][125]. Кроме того, в связи с резким снижением уровня прогестерона (природного антагониста альдостерона) происходит реактивация ренин-ангиотензин-альдостероновой системы (РААС) с такими последствиями, как задержка жидкости, повышение АД [130].

Выделяют следующие специфичные для женщин патофизиологические характеристики АГ [131]:

– тесная связь ожирения с АГ;

– связь гинекологических нарушений (ановуляция, пролиферативные гинекологические заболевания) и неблагоприятного течения беременности преэклампсия, гестационный СД) с риском кардиометаболических нарушений и АГ;

– кардиовазопротективный эффект (в том числе вазодилатирующий) физиологического для репродуктивного возраста уровня эстрогена;

– фармакологическое использование эстрогена при наличии сформировавшейся дисфункции эндотелия может способствовать увеличению АД и риска развития ССЗ, введение экзогенных эстрогенов в дозировках, применяемых для МГТ, не оказывает негативное влияние на АД;

– прогестерон способствует лептин-опосредованной дисфункции эндотелия у женщин с ожирением до наступления менопаузы;

– более выражена чувствительность к натрию;

– более высокая частота развития воспалительных заболеваний, связанных с АГ и ССЗ.

В постменопаузе у женщин наблюдается более быстрое, чем у мужчин такого же возраста, увеличение жесткости артерий. У женщин пожилого возраста отмечается более высокая, чем у мужчин, ригидность аорты. Это, по-видимому, способствует развитию изолированной систолической АГ, неконтролируемой АГ, сердечной недостаточности с сохраненной фракцией выброса левого желудочка (ФВ ЛЖ), аортальному стенозу, что чаще встречается у женщин [132][133].

Установлено, что менопауза удваивает риск развития АГ даже после поправки на возраст и ИМТ [134]. Хотя МГТ содержит эстрогены, нет убедительных доказательств того, что АД будет значительно повышаться у женщин в менопаузе с АГ или без нее [135]. Однако после начала МГТ необходимо рекомендовать регулярное измерение АД для подтверждения сохраняющегося нормального АД или контроля уровня АД при антигипертензивной терапии [136][137]. В случае неконтролируемой АГ МГТ следует прекратить. Решение об отмене МГТ целесообразно принимать совместно с кардиологом.

Ключевые положения:

– МГТ может быть назначена при условии контроля АД.

– МГТ не назначается для первичной или вторичной профилактики ССЗ.

## 7.3. Курение

Курение значительно увеличивает опасность артериальных ССО и является ФР развития злокачественных новообразований.

Курение не является ФР развития ВТЭО при МГТ (включая комбинированную пероральную МГТ). Несмотря на то что курение не служит основанием для отказа от МГТ, в том числе комбинированными пероральными препаратами, необходимо соблюдать осторожность при назначении МГТ курильщицам, информировать их о рисках для здоровья, связанных с курением, и настаивать на прекращении курения. Женщинам, продолжающим курить, несмотря на все предостережения, следует сообщить, что курение помимо всех своих других негативных эффектов, может также поставить под угрозу успех МГТ [57][138–140].

Ключевые положения:

– Необходимо информировать женщин о рисках для здоровья, связанных с курением, и настаивать на его прекращении.

– У курящих женщин решение о возможности применения МГТ следует принимать с учетом совокупности всех ФР.

## РАЗДЕЛ 8. МГТ В ОСОБЫХ КЛИНИЧЕСКИХ СИТУАЦИЯХ

## 8.1. Атеросклероз периферических артерий

Среди женщин в возрасте 45–49 лет распространенность атеросклероза периферических артерий составляет 4,89%, в возрасте 50–55 лет — 5,73%, в возрасте 56–60 лет — 6,73%. Менопауза увеличивает риск развития каротидного атеросклероза в 2 раза [141]. Преждевременная и ранняя менопауза связана с увеличением объема и распространенности атеросклеротических бляшек [142].

Применение монотерапии эстрогенами у женщин в постменопаузе в течение года снижает риск развития атеросклероза периферических артерий на 52%, как показано в наблюдательном исследовании Rotterdam study [142]. В другом наблюдательном исследовании определено, что МГТ независимо от ее выбора снижает риск развития атеросклероза периферических артерий на 20% [144]. В последних наблюдательных исследованиях показан положительное действие эстрогенов на толщину интимы-медии сонных артерий только в ранний период после менопаузы (до 6 лет), в более поздний период, особенно после 10 лет, эстрогены способствуют прогрессированию атеросклероза [145].

МГТ не снижает риск прогрессирования атеросклероза у женщин с установленным атеросклеротическими ССЗ [146].

У больных ИБС в РКИ HERS и HERSII (Heart and Estrogen/progestin Replacement Study) комбинированная пероральная МГТ не обеспечила статистически значимого снижения числа осложнений, связанных с атеросклерозом периферических артерий [89][147].

В описательном обзоре R.S. Davies и соавт. [148] в качестве механизма, положительного влияния МГТ на течение периферического атеросклероза, обсуждается снижение уровня ХС ЛНП в сыворотке крови, повышение уровня ХС ЛВП и положительное воздействие на функцию эндотелия.

## 8.2. Хроническая сердечная недостаточность

В Российской Федерации по данным популяционного исследования ЭПОХА-ХСН распространенность хронической сердечной недостаточности (ХСН) у женщин в возрасте 50 лет составляет 12,2%, в возрасте 60 лет — 26,2%, преимущественно с сохраненной ФВ ЛЖ [149]. Пятилетняя выживаемость больных с ХСН составляет не более 50% [150].

Ранняя постменопауза увеличивает риск развития ХСН на 33%, как выявлено в метаанализе трех наблюдательных исследований [151].

В РКИ после 10 лет лечения выявлено, что женщины, получающие пероральную терапию эстрогенами или комбинированную МГТ, назначенную в первые 7 мес в среднем после менопаузы, имели значительно ниже риск смерти, развития ХСН, ИМ без увеличения риска развития рака, ВТЭО или инсульта [152].

Пероральная и трансдермальная терапия эстрогенами, а также комбинированная МГТ у пациенток в возрасте 50 лет и старше с ХСН III–IV функционального класса и ФВ ЛЖ ≤35% неишемической этиологии обеспечила статистически значимое снижение общей смертности на 40%, как продемонстрировано в анализе в подгруппах РКИ BEST (Beta-Blocker Evaluation of Survival Trial) [152].

Последний метаанализ 6 РКИ и наблюдательных исследований, в который были включены 25 047 женщин, не выявил статистической связи между любым вариантом МГТ и риском развития ХСН у женщин в постменопаузе. При этом снижение смертности от всех причин на 35% наблюдалось среди пациенток с ХСН, которые получали МГТ. В анализе возрастных подгрупп не замечено существенного изменения риска развития ХСН [153].

Анализ в подгруппах РКИ WHI (Women’s Health Initiative) показал, что монотерапия пероральными эстрогенами и комбинированная МГТ не увеличивает риск госпитализаций, связанных с ХСН, независимо от ФВ ЛЖ и возраста женщины при назначении МГТ [154].

По данным мониторинга за выпиской рецептов и визитами к врачу системы Medicare среди 10 млн женщин в возрасте 65 лет и старше, монотерапия эстрогенами статистически значимо снижала риск развития ХСН на 5%, комбинированная МГТ — на 4% [155].

## 8.3. Фибрилляция предсердий

Известно, что женщины во всех возрастных группах имеют более низкую распространенность фибрилляции предсердий (ФП) по сравнению с мужчинами, но смертность от всех причин у женщин выше: ФП независимо связана с двукратным увеличением риска смерти у женщин по сравнению с 1,5-кратным увеличением риска смерти у мужчин [156]. В наблюдательном исследовании ATRIA ежегодная частота развития тромбоэмболических осложнений у пациентов с ФП, не принимающих варфарин, составила 3,5% у женщин по сравнению с 1,8% у мужчин [156]. До 2024 г. считалось, что женщины с дополнительными ФР развития инсульта, особенно в старшем возрасте (>65 лет), подвергаются большему риску развития инсульта, даже если принимают антикоагулянтную терапию, в то время как риск кровотечения при антикоагуляции у мужчин и женщин был одинаковым [157]. В новых рекомендациях Европейского общества кардиологов принято решение о том, что женский пол является лишь возрастным модификатором риска развития инсульта, а не независимым биологическим ФР [157]. При этом у женщин с ФП более выражены симптоматика и тяжесть инсультов.

В постменопаузе на 82% увеличивается риск формирования ФП [158]. Показано, что эстроген оказывает как защитное, так и проаритмическое действие на сердце [159].

Данные наблюдательного исследования BiomarCaRE Consortium в Европе продемонстрировали, что у женщин в постменопаузе (средний возраст 49,2 года) распространенность ФП составила 4,4%, что было взаимосвязано с увеличением риска развития инсульта на 42%, ИМ на 78%, а частота смертельных исходов увеличивалась более чем в 3,5 раза [159].

По данным анализа в подгруппах РКИ WHI (Women’s Health Initiative) и наблюдательных исследований комбинированная МГТ, монотерапия пероральными эстрогенами, применение тиболона увеличивают риск развития ФП [158][161–163].

Исследования, проведенные в последние годы, демонстрируют противоречивые данные в этом вопросе. В ретроспективном наблюдательном исследовании продемонстрировано, что применение МГТ, за исключением использования монотерапии эстрадиолом в комбинации его с прогестином, увеличивало риск развития ФП у женщин в менопаузе [164]. У женщин, принимающих ранее эстрадиол в сочетании с прогестином, отмечалось снижение риска формирования ФП с поправкой на факторы риска (p=0,027). Продолжающаяся МГТ представляла повышенный риск развития ФП. Степень риска варьировала в зависимости от конкретного типа эстрогена и прогестинов при сочетанном назначении. Результаты исследования указывают на то, что в отношении риска развития ФП пероральная эстрадиолсодержащая МГТ превосходит МГТ, содержащую пероральный лошадиный конъюгированный эстроген или тиболон.

Появились новые сведения мониторинга за выпиской рецептов и визитами к врачу системы Medicare среди 10 млн женщин в возрасте 65 лет и старше, в которых определено, что монотерапия эстрогенами и пероральная комбинированная МГТ статистически значимо снижала риск развития ФП на 4% независимо от вида МГТ [155].

Вклад локальных форм эстрогенов в развитие ФП у женщин в период менопаузы не определен.

## 8.4. Патология клапанов сердца

Возможность назначения пероральной МГТ у женщин в пери- и постменопаузе с патологией клапанов определяется наличием осложнений:

– при ФП и тромбах в камерах сердца — МГТ противопоказана;

– при ХСН неишемической этиологии и в отсутствие осложнений МГТ может быть назначена в рамках междисциплинарного консилиума [165].

## 8.5. Легочная гипертензия

Половые различия влияют не только на распространенность легочной гипертензии (ЛГ) независимо от ее патогенеза, но и на ее тяжесть, реакцию на лечение и результаты выживаемости [166]. Хотя женщины более восприимчивы к развитию ЛГ, у них также отмечаются лучшая реакция на лечение и более высокая выживаемость по сравнению с мужчинами [166]. Эти различия были названы «эстрогенный парадокс» с положительным влиянием эстрогена на течение и прогноз [167].

Результаты исследований, касающиеся использования МГТ при ЛГ, противоречивы. В экспериментальных исследованиях выявлено, что МГТ может улучшить состояние правых отделов сердца и снизить давление в легочной артерии [168]. Тем не менее крупное одноцентровое исследование, проведенное в 2020 г., не выявило существенных различий в эндо- или экзогенном воздействии половых гормонов в отношении развития и прогрессирования ЛГ [168].

## РАЗДЕЛ 9. МИГРЕНЬ

Мигрень в популяции стран Европы и США встречается у 17% женщин и 8% мужчин. Взрослые женщины болеют в 2,5–3 раза чаще, чем мужчины [169].

Установлена зависимость частоты приступов мигрени от колебаний уровней половых стероидных гормонов.

Выделяют две основные формы мигрени: без ауры (до 80% всех случаев) и с аурой (20% всех случаев) — аура представляет собой преходящие неврологические нарушения (зрительные, чувствительные и/или речевые симптомы), предшествующие головной боли [170]. Мигрень с аурой сопряжена с двукратным увеличением риска развития инсульта [171].

При подозрении на мигрень у женщин в постменопаузе необходима консультация невролога для исключения другой патологии.

Поскольку данные о взаимосвязи между МГТ и мигренью отсутствуют, нельзя сделать вывод, что мигрень служит противопоказанием к МГТ [172].

Мигрень без ауры совместима практически с любыми вариантами и режимами МГТ. Непрерывная комбинированная МГТ в меньшей степени провоцирует головную боль, чем циклический режим, особенно при использовании низких доз эстрогена [173]. Мигрень с аурой не служит противопоказанием к МГТ. При мигрени с аурой следует использовать минимальную дозу эстрогенного компонента, биодентичного натуральному, которая эффективно контролирует вазомоторные симптомы [172]. Возможно использование трансдермальных эстрогенов в качестве монотерапии, которые обеспечивают стабильную плазменную концентрацию эстрадиола без резких пиковых суточных колебаний и позволяют минимизировать риск возникновения приступов мигрени или в комбинации с внутриматочной системой, содержащей 52 мг левоноргестрела микронизированного (ЛНГ-ВМС) [172][174].

## ЗАКЛЮЧЕНИЕ

Показания и противопоказания к назначению менопаузальной гормональной терапии определяются актуальными клиническими рекомендациями и инструкциями к конкретным препаратам.

Свод критериев приемлемости назначения менопаузальной гормональной терапии пациенткам с сердечно-сосудистыми и метаболическими заболеваниями приведен в Приложении 1. Для унификации рекомендаций были определены следующие категории в соответствии с международной номенклатурой ВОЗ [65].

КАТЕГОРИЯ 1 — нет ограничений для использования МГТ;

КАТЕГОРИЯ 2 — польза от применения МГТ превышает риски;

КАТЕГОРИЯ 3 — возможные риски превышают пользу;

КАТЕГОРИЯ 4 — не рекомендуется применение МГТ.

При обращении женщины с жалобами на приливы, потливость, сердцебиения врачу необходимо провести опрос с целью выявления взаимосвязи жалоб с возможными климактерическими нарушениями. Опрос должен включать сведения о дате последней самостоятельной менструации, нарушении регулярности менструального цикла и текущем приеме гормональной контрацепции или менопаузальной гормональной терапии. В случае подозрения на связь жалоб с климактерическими расстройствами необходимо направить женщину на консультацию к акушеру-гинекологу.

Назначение менопаузальной гормональной терапии, коррекция дозы, смена лекарственного средства, прекращение менопаузальной гормональной терапии, ежегодный динамический контроль за эффективностью/переносимостью лечения, актуализацию целей терапии и оценку баланса польза/риск проводит акушер-гинеколог (Приложения 2 и 3).

При выявлении/подозрении на наличие нежелательных явлений, ассоциированных с приемом менопаузальной гормональной терапии врачом негинекологического профиля пациентке должна быть рекомендована консультация акушера-гинеколога.

При выявлении/подозрении на наличие факторов риска развития сердечно-сосудистых заболеваний и сердечно-сосудистых и метаболических заболеваний акушерами-гинекологами пациентке должна быть рекомендована консультация врача терапевтического профиля.

## ДОПОЛНИТЕЛЬНАЯ ИНФОРМАЦИЯ

Источники финансирования. Работа выполнена по инициативе авторов без привлечения финансирования

Конфликт интересов. Авторы декларируют отсутствие явных и потенциальных конфликтов интересов, связанных с содержанием настоящей статьи.

Участие авторов. Все авторы одобрили финальную версию статьи перед публикацией, выразили согласие нести ответственность за все аспекты работы, подразумевающую надлежащее изучение и решение вопросов, связанных с точностью или добросовестностью любой части работы.

## ПРИЛОЖЕНИЕ 1

**Table:** Таблица 6. Критерии приемлемости назначения МГТ КАТЕГОРИЯ 1 — нет ограничений для использования МГТ; КАТЕГОРИЯ 2 — польза от применения МГТ превышает риски; КАТЕГОРИЯ 3 — возможные риски превышают пользу; КАТЕГОРИЯ 4 — не рекомендуется применение МГТ. НП — неприменимо из-за отсутствия данных; МГТ — менопаузальная гормональная терапия; СД — сахарный диабет; ИМТ — индекс массы тела; ТЭЛА — тромбоэмболия легочной артерии; ТГВ — тромбоз глубоких вен; ВТЭО — венозные тромбоэмболические осложнения; ИБС — ишемическая болезнь сердца; ССЗ — сердечно-сосудистые заболевания; ТГ — триглицериды; АД — артериальное давление.

	Комбинированная МГТ	Монотерапия эстрогенами	Тиболон	Локальная МГТЭстриол или прастерон	Примечание
перорально	трансдермально	перорально	трансдермально
Нарушения углеводного обмена
СД	2	2	2	2	НП	1	
ИМТ, кг/м²18,5–24,925–29,930–34,9≥35	112*/3**3***	112*/3**3***	112*/3**3***	112*/3**3***	НПНПНПНП	1111	2* — отсутствие кардиометаболических заболеваний, связанных с ожирением или нахождение под наблюдением у профильного специалиста, заболевания в стадии компенсации (Приложение 2).3** — наличие одного или нескольких кардиометаболических заболеваний, ассоциированных с ожирением (Приложение 2).***Нет данных по ИМТ >35 кг/м²
Венозные тромбозы и/или ТЭЛА
Острый ТГВ/ТЭЛА	4	4	4	4	4	1	Под острыми ТГВ/ТЭЛА понимается период, требующий использования полной лечебной дозой антикоагулянта (основная фаза антикоагулянтной терапии, первые 3–6 мес)
ТГВ/ТЭЛА в анамнезе	4	3	4	3	4	1	При тяжелых менопаузальных симптомах во время лечения антикоагулянтами у отдельных больных можно рассмотреть трансдермальную или ультранизкодозированную пероральную МГТ; в большинстве случаев МГТ не следует использовать после отмены антикоагулянтов
Тромбоз поверхностных вен (острый или в анамнезе)	3	3	3	3	НП	1	
Нетромботические хронические заболевания вен
Нетромботические хронические заболевания вен (варикозное расширение вен, ретикулярные вены, телеангиэктазии нижних конечностей)	1	1	1	1	1	1	
Тромбофилии
Бессимптомная тромбофилия с высоким риском развития ВТЭО (дефицит протеина S, дефицит протеина С, дефицит антитромбина)	4	4	4	4	НП	НП	
Бессимптомная тромбофилия лейденская мутация гена фактора V, мутация гена протромбинa G20210A, высокий уровень фактора свертывания крови VIII	2	2	2	2	НП	1	Необходимо учитывать ранее выявленную тромбофилию, обследование на тромбофилию перед назначением МГТ не требуется.Решение о возможности назначения и составе МГТ следует принимать индивидуально с учетом сведений о наличии ранее выявленной бессимптомной тромбофилии, тяжести менопаузальных симптомов, наличия дополнительных факторов риска развития ВТЭО, а также указания определенных тромбофилий в перечне противопоказаний в инструкции к конкретному препарату для МГТ.По имеющимся данным, трансдермальные препараты для МГТ не повышают риск венозных тромбозов у пациенток с бессимптомной тромбофилией
Антифосфолипидный синдром	4	3	4	3	4	1	Возможность МГТ не исключена у женщин с низкой или умеренной активностью заболевания, не имеющих дополнительных факторов риска венозных тромбозов
Семейный анамнез тромбозов	2	2	2	2	2	1	Наличие родственника I степени родства, перенесшего венозный или антериальный тромбоз в возрасте моложе 50 лет
Хирургические вмешательства и острые нехирургические заболевания с госпитализацией
Хирургическое вмешательство	1	1	1	1	1	1	Перед хирургическим вмешательством необходима оценка риска развития ТГВ/ТЭЛА в послеоперационном периоде по шкале Каприни. Рекомендуется при оценке риска развития послеоперационных ТГВ/ТЭЛА учитывать проведение МГТ как 1 дополнительный балл по шкале Каприни. Отмена МГТ при хирургических вмешательствах не требуется. Профилактика венозных тромбозов антикоагулянтами должна проводиться в соответствии с определенной по шкале Каприни категорией риска развития ТГВ/ТЭЛА.
Острые нехирургические заболевания, требующие госпитализации	1	1	1	1	1	1	При госпитализации необходима оценка риска развития ТГВ/ТЭЛА по рекомендуемым шкалам (например, шкала Padua). Рекомендуется при оценке риска развития послеоперационных ТГВ/ТЭЛА учитывать проведение МГТ как 1 дополнительный балл. Отмена МГТ при острых нехирургических заболеваниях, требующих госпитализации, не входящих в состав противопоказаний к МГТ, не требуется. Профилактика венозных тромбозов антикоагулянтами должна проводиться в соответствии с определенной по шкале категорией риска развития ТГВ/ТЭЛА
Атеросклеротические сердечно-сосудистые заболевания
ИБС	3	3	3	3	НП	1	При наличии ИБС начало МГТ не рекомендовано. У пациенток с развившейся в процессе терапии ИБС, настроенных на продолжение МГТ, вопрос о ее отмене должен быть решен индивидуально кардиологом и гинекологом совместно
Инфаркт миокарда (острый или в анамнезе)	4	4	4	4	4	1	
Нарушение мозгового кровообращения, включая транзиторную ишемическую атаку (острое или в анамнезе)	4	4	4	4	4	1	
Факторы риска развития ССЗ
Гиперлипидемия (кроме гипертриглицеридемии)	1	1	1	1	1	1	
Гипертриглицеридемия	3	2	3	2	2	1	При уровне ТГ >4,5 ммоль/л не рекомендовано начало МГТ, требуется коррекция уровня ТГ
Артериальная гипертензия	1	1	1	1	1	1	МГТ может быть назначена при условии контроля АД
Курение	2/3*	2/3*	2/3*	2/3*	2/3*	1	3*При наличии любых других факторов риска развития ССЗ или ССЗ направление на консультацию терапевта/кардиолога
Другие заболевания/состояния
Атеросклероз периферических артерий	3	3	3	НП	НП	НП	
Хроническая сердечная недостаточность (неишемического генеза)	3	3	3	НП	НП	НП	
Фибрилляция предсердий	4	4	4	4	4	НП	
Легочная гипертензия	4	4	4	НП	НП	НП	Доказательства получены в одном наблюдательном исследовании
Мигрень без ауры	1	1	1	1	НП	1	
Мигрень с аурой	2	1	2	1	НП	1	

## ПРИЛОЖЕНИЕ 2

**Table:** Таблица 7. Кардиометаболические заболевания, связанные с ожирением АД — артериальное давление ТГ — триглицериды; ХС ЛВП — холестерин липопротеидов высокой плотности; ОХС — общий холестерин; ХС ЛНП — холестерин липопротеидов низкой плотности; АлАТ — аланинаминотрансфераза, АсАТ — аспартатаминотрансфераза; ГГТ — гамма-глутамилтранспептидаза.

Нарушения, связанные с ожирением	Идентификация на основе информации, полученной при первоначальной оценке
Метаболический синдром	Окружность талии, АД, уровень в крови ТГ, ХС ЛВП, глюкозы натощак
Предиабет	Нарушение гликемии натощак/нарушение толерантности к глюкозе
Сахарный диабет 2-го типа	Уровень глюкозы натощак, гликированный гемоглобин
Дислипидемия	Уровень ОХС, ХС ЛНП, ХС ЛВП, ТГ
Артериальная гипертензия	Систолическое и диастолическое АД
Неалкогольная жировая болезнь печени	Функциональные пробы печени (определение активности АлАТ, АсАТ, ГГТ в крови)

## ПРИЛОЖЕНИЕ 3

**Figure:**
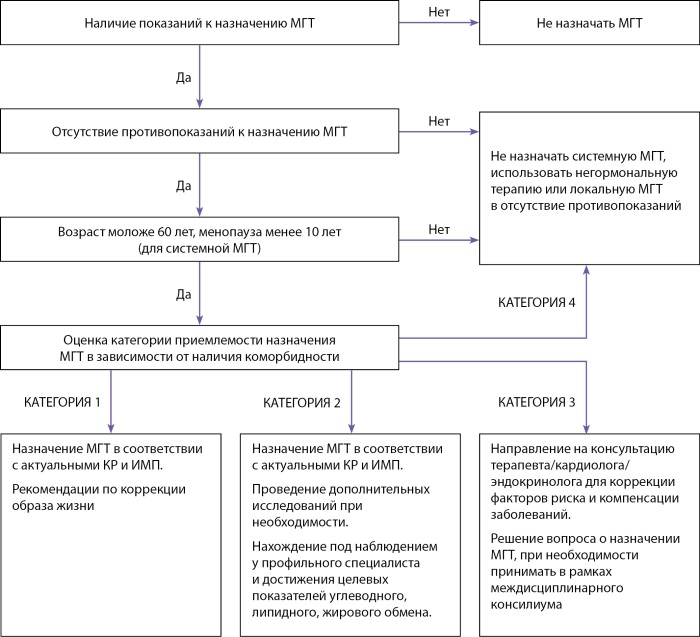
Рисунок 2. Алгоритм принятия решения о назначении МГТ МГТ — менопаузальная гормональная терапия; КР — клинические рекомендации; ИМП —инструкции медицинских препаратов

## ПРИЛОЖЕНИЕ 4

**Figure:**
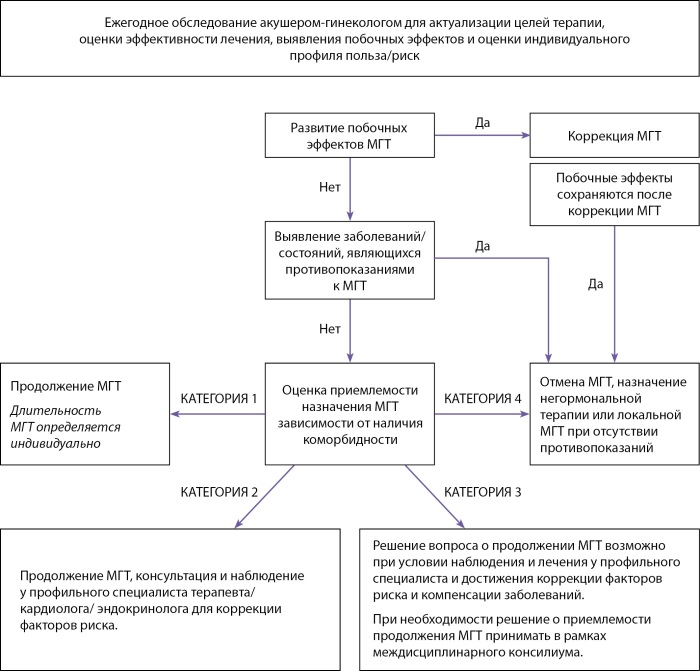
Рисунок 3. Алгоритм принятия решения об отмене МГТ МГТ — менопаузальная гормональная терапия.

## References

[cit1] Pravitel'stvo Rossiiskoi Federatsii. Rasporyazhenie Pravitel'stva Rossiiskoi Federatsii ot 29.12.2022 № N 4356-p “Ob utverzhdenii Natsional'noi strategii deistvii v interesakh zhenshchin na 2023—2030 gody”. Dostupno na: https://www.consultant.ru/document/cons_doc_LAW_.

[cit2] Ulumbekova G.E., Khudova I.Yu. Otsenka demograficheskogo, sotsial'nogo i ekonomicheskogo effekta pri prieme menopauzal'noi gormonal'noi terapii. ORGZDRAV: novosti, mneniya, obucheniya. Vestnik VShOUZ. 2020;6(4(22)):23–53. doi: https://doi.org/10.24411/2411-8621-2020-14002

[cit3] Lambrinoudaki Irene, Armeni Eleni, Goulis Dimitrios, Bretz Silvia, Ceausu Iuliana, Durmusoglu Fatih, Erkkola Risto, Fistonic Ivan, Gambacciani Marco, Geukes Marije, Hamoda Haitham, Hartley Caiomhe, Hirschberg Angelica Lindén, Meczekalski Blazej, Mendoza Nicolas, Mueck Alfred, Smetnik Antonina, Stute Petra, van Trotsenburg Mick, Rees Margaret (2022). Menopause, wellbeing and health: A care pathway from the European Menopause and Andropause Society. Maturitas.

[cit4] Ministerstvo zdravookhraneniya Rossiiskoi Federatsii. Klinicheskie rekomendatsii. Menopauza i klimaktericheskoe sostoyanie u zhenshchiny. 2021. Dostupno na: https://cr.minzdrav.gov.ru/previewcr/117_2

[cit5] Harlow Siobán D., Gass Margery, Hall Janet E., Lobo Roger, Maki Pauline, Rebar Robert W., Sherman Sherry, Sluss Patrick M., de Villiers Tobie J. (2012). Executive summary of the Stages of Reproductive Aging Workshop + 10. Menopause.

[cit6] Drewe Juergen, Bucher Kathleen A, Zahner Catherine (2015). A systematic review of non-hormonal treatments of vasomotor symptoms in climacteric and cancer patients. SpringerPlus.

[cit7] Schnatz Peter F., Romegialli Alison, Abrantes Jessica, Marakovits Kimberly, Cunningham David, O'Sullivan David M. (2008). The North American Menopause Society. Menopause.

[cit8] Paramsothy Pangaja, Harlow Siobán D., Nan Bin, Greendale Gail A., Santoro Nanette, Crawford Sybil L., Gold Ellen B., Tepper Ping G., Randolph John F. (2016). Duration of the menopausal transition is longer in women with young age at onset: the multiethnic Study of Women's Health Across the Nation. Menopause.

[cit9] Ministerstvo zdravookhraneniya Rossiiskoi Federatsii. Menopauzal'naya gormonoterapiya i sokhranenie zdorov'ya zhenshchin v zrelom vozraste. Klinicheskie rekomendatsii. 2015. Dostupno na: https://www.consultant.ru/document/cons_doc_LAW_320073

[cit10] Schoenaker Danielle AJM, Jackson Caroline A, Rowlands Jemma V, Mishra Gita D (2014). Socioeconomic position, lifestyle factors and age at natural menopause: a systematic review and meta-analyses of studies across six continents. International Journal of Epidemiology.

[cit11] Freeman Ellen W., Sammel Mary D., Sanders Richard J. (2014). Risk of long-term hot flashes after natural menopause. Menopause.

[cit12] Costanian C., Zangiabadi S., Bahous S. A., Deonandan R., Tamim H. (2020). Reviewing the evidence on vasomotor symptoms: the role of traditional and non-traditional factors. Climacteric.

[cit13] Prior JC. Progesterone for Symptomatic Perimenopause Treatment - Progesterone politics, physiology and potential for perimenopause. Facts, Views & Vision in ObGyn. 2011;3(2):109–20. PMID: 24753856 PMC3987489 24753856

[cit14] Santoro Nanette, Epperson C. Neill, Mathews Sarah B. (2015). Menopausal Symptoms and Their Management. Endocrinology and Metabolism Clinics of North America.

[cit15] Mel'nichenko Galina A., Belaya Zhanna E., Rozhinskaya Lyudmila Ya., Toroptsova Natalia V., Alekseeva Lyudmila I., Biryukova Elena V., Grebennikova Tatiana A., Dzeranova Larisa K., Dreval Aleksandr V., Zagorodniy Nikolay V., Il'yin Aleksandr V., Kryukova Irina V., Lesnyak Ol’ga M., Mamedova Elizaveta O., Nikitinskaya Oksana A., Pigarova Ekaterina A., Rodionova Svetlana S., Skripnikova Irina A., Tarbaeva Natalia V., Farba Leonid Ya., Tsoriev Timur T., Chernova Tatiana O., Yureneva Svetlana V., Yakushevskaya Oksana V., Dedov Ivan I. (2018). Russian federal clinical guidelines on the diagnostics, treatment, and prevention of osteoporosis. Problems of Endocrinology.

[cit16] Muka Taulant, Oliver-Williams Clare, Colpani Veronica, Kunutsor Setor, Chowdhury Susmita, Chowdhury Rajiv, Kavousi Maryam, Franco Oscar H. (2016). Association of Vasomotor and Other Menopausal Symptoms with Risk of Cardiovascular Disease: A Systematic Review and Meta-Analysis. PLOS ONE.

[cit17] Baber R. J., Panay N., Fenton A. (2016). 2016 IMS Recommendations on women’s midlife health and menopause hormone therapy. Climacteric.

[cit18] Kaufman Melissa R., Ackerman A. Lenore, Amin Katherine A., Coffey Marge, Danan Elisheva, Faubion Stephanie S., Hardart Anne, Goldstein Irwin, Ippolito Giulia M., Northington Gina M., Powell Charles R., Rubin Rachel S., Westney O. Lenaine, Wilson Tracey S., Lee Una J. (2025). The AUA/SUFU/AUGS Guideline on Genitourinary Syndrome of Menopause. Journal of Urology.

[cit19] (2020). The 2020 genitourinary syndrome of menopause position statement of The North American Menopause Society. Menopause.

[cit20] Rueda C., Osorio A. M., Avellaneda A. C., Pinzón C. E., Restrepo O. I. (2017). The efficacy and safety of estriol to treat vulvovaginal atrophy in postmenopausal women: a systematic literature review. Climacteric.

[cit21] Hirschberg Angelica Lindén, Bitzer Johannes, Cano Antonio, Ceausu Iuliana, Chedraui Peter, Durmusoglu Fatih, Erkkola Risto, Goulis Dimitrios G., Kiesel Ludwig, Lopes Patrice, Pines Amos, van Trotsenburg Mick, Lambrinoudaki Irene, Rees Margaret (2021). Topical estrogens and non-hormonal preparations for postmenopausal vulvovaginal atrophy: An EMAS clinical guide. Maturitas.

[cit22] Weidlinger S., Schmutz C., Janka H., Gruetter C., Stute P. (2021). Sustainability of vaginal estrogens for genitourinary syndrome of menopause – a systematic review. Climacteric.

[cit23] Matarazzo Maria Grazia, Sarpietro Giuseppe, Fiorito Debora, Di Pasqua Salvatore, Ingrassano Simona, Panella Marco Marzio, Cianci Antonio, Caruso Salvatore (2021). Intravaginal 6.5 mg prasterone administration in postmenopausal women with overactive bladder syndrome: A pilot study. European Journal of Obstetrics & Gynecology and Reproductive Biology.

[cit24] Duarte Pablo Romero, Maroto Martín María Teresa, Mar Martín Moya María del, Prados Pedro Abad (2022). Quality of life analysis measured with the Cervantes 16 scale in treated menopausal women with genitourinary syndrome. Journal of Comparative Effectiveness Research.

[cit25] Labrie Fernand (2018). Intracrinology and menopause: the science describing the cell-specific intracellular formation of estrogens and androgens from DHEA and their strictly local action and inactivation in peripheral tissues. Menopause.

[cit26] Labrie Fernand, Archer David F., Koltun William, Vachon Andrée, Young Douglas, Frenette Louise, Portman David, Montesino Marlene, Côté Isabelle, Parent Julie, Lavoie Lyne, BSc Adam Beauregard, Martel Céline, Vaillancourt Mario, Balser John, Moyneur Érick (2018). Efficacy of intravaginal dehydroepiandrosterone (DHEA) on moderate to severe dyspareunia and vaginal dryness, symptoms of vulvovaginal atrophy, and of the genitourinary syndrome of menopause. Menopause.

[cit27] Labrie Fernand, Archer David F., Martel Céline, Vaillancourt Mario, Montesino Marlene (2017). Combined data of intravaginal prasterone against vulvovaginal atrophy of menopause. Menopause.

[cit28] Cappola Anne R, Auchus Richard J, El-Hajj Fuleihan Ghada, Handelsman David J, Kalyani Rita R, McClung Michael, Stuenkel Cynthia A, Thorner Michael O, Verbalis Joseph G (2023). Hormones and Aging: An Endocrine Society Scientific Statement. The Journal of Clinical Endocrinology & Metabolism.

[cit29] (2025). Joint position of Russian experts on the clinical significance of hyperandrogenism in early postmenopausal women. Akusherstvo i ginekologiia.

[cit30] Gluvic Zoran, Zaric Bozidarka, Resanovic Ivana, Obradovic Milan, Mitrovic Aleksandar, Radak Djordje, Isenovic Esma (2016). Link between Metabolic Syndrome and Insulin Resistance. Current Vascular Pharmacology.

[cit31] (2015). Metabolic Syndrome and Menopause. Advances in Clinical Chemistry.

[cit32] Hirschberg Angelica Lindén (2023). Hyperandrogenism and Cardiometabolic Risk in Pre- and Postmenopausal Women—What Is the Evidence?. The Journal of Clinical Endocrinology & Metabolism.

[cit33] Hirschberg Angelica Lindén (2022). Approach to Investigation of Hyperandrogenism in a Postmenopausal Woman. The Journal of Clinical Endocrinology & Metabolism.

[cit34] Frederiksen Hanne, Johannsen Trine Holm, Andersen Stine Ehlern, Petersen Jørgen Holm, Busch Alexander Siegfried, Ljubicic Marie Lindhardt, Fischer Margit Bistrup, Upners Emmie N., Hagen Casper P., Main Katharina M., Aksglaede Lise, Jørgensen Niels, Lund Kårhus Line, Linneberg Allan, Andersson Anna-Maria, Flück Christa E., Juul Anders (2024). Sex- and age-specific reference intervals of 16 steroid metabolites quantified simultaneously by LC-MS/MS in sera from 2458 healthy subjects aged 0 to 77 years. Clinica Chimica Acta.

[cit35] Torréns Javier I., Sutton-Tyrrell Kim, Zhao Xinhua, Matthews Karen, Brockwell Sarah, Sowers MaryFran, Santoro Nanette (2009). Relative androgen excess during the menopausal transition predicts incident metabolic syndrome in midlife women. Menopause.

[cit36] Alemany Marià (2022). The Roles of Androgens in Humans: Biology, Metabolic Regulation and Health. International Journal of Molecular Sciences.

[cit37] Galmés-Pascual Bel M., Martínez-Cignoni Melanie Raquel, Morán-Costoya Andrea, Bauza-Thorbrügge Marco, Sbert-Roig Miquel, Valle Adamo, Proenza Ana M., Lladó Isabel, Gianotti Magdalena (2020). 17β-estradiol ameliorates lipotoxicity-induced hepatic mitochondrial oxidative stress and insulin resistance. Free Radical Biology and Medicine.

[cit38] Nestler John E., Jakubowicz Daniela J., Falcon de Vargas Aida, Brik Carlos, Quintero Nitza, Medina Francisco (2014). Insulin Stimulates Testosterone Biosynthesis by Human Thecal Cells from Women with Polycystic Ovary Syndrome by Activating Its Own Receptor and Using Inositolglycan Mediators as the Signal Transduction System^1^. The Journal of Clinical Endocrinology & Metabolism.

[cit39] Xing Chuan, Zhang Jiaqi, Zhao Han, He Bing (2022). Effect of Sex Hormone-Binding Globulin on Polycystic Ovary Syndrome: Mechanisms, Manifestations, Genetics, and Treatment. International Journal of Women's Health.

[cit40] Dicker A., Rydén M., Näslund E., Muehlen I. E., Wirén M., Lafontan M., Arner P. (2004). Effect of testosterone on lipolysis in human pre-adipocytes from different fat depots. Diabetologia.

[cit41] Zang Hong, Rydén Mikael, Wåhlen Kerstin, Dahlman-Wright Karin, Arner Peter, Hirschberg Angelica Lindén (2007). Effects of testosterone and estrogen treatment on lipolysis signaling pathways in subcutaneous adipose tissue of postmenopausal women. Fertility and Sterility.

[cit42] Grundy Scott M. (2007). Metabolic Syndrome: A Multiplex Cardiovascular Risk Factor. The Journal of Clinical Endocrinology & Metabolism.

[cit43] Hu G. (2016). Gender difference in all-cause and cardiovascular mortality related to hyperglycaemia and newly-diagnosed diabetes. Diabetologia.

[cit44] Ministerstvo zdravookhraneniya Rossiiskoi Federatsii. Klinicheskie rekomendatsii. Sakharnyi diabet 2 tipa u vzroslykh. 2022. Dostupno na: https://cr.minzdrav.gov.ru/preview-cr/290_2

[cit45] Zhang Lulu, Bao Lei, Li Yuqian, Wang Chongjian, Dong Xiaokang, Abdulai Tanko, Yang Xiu, Fan Mengying, Cui Songyang, Zhou Wen, Mao Zhenxing, Huo Wenqian, Wei Dandan, Li Linlin (2020). Age at menopause, body mass index, and risk of type 2 diabetes mellitus in postmenopausal Chinese women: The Henan Rural Cohort study. Nutrition, Metabolism and Cardiovascular Diseases.

[cit46] Opoku Albert A., Abushama Mandy, Konje Justin C. (2023). Obesity and menopause. Best Practice & Research Clinical Obstetrics & Gynaecology.

[cit47] Vishram Julie K. K., Borglykke Anders, Andreasen Anne H., Jeppesen Jørgen, Ibsen Hans, Jørgensen Torben, Palmieri Luigi, Giampaoli Simona, Donfrancesco Chiara, Kee Frank, Mancia Giuseppe, Cesana Giancarlo, Kuulasmaa Kari, Salomaa Veikko, Sans Susana, Ferrieres Jean, Dallongeville Jean, Söderberg Stefan, Arveiler Dominique, Wagner Aline, Tunstall-Pedoe Hugh, Drygas Wojciech, Olsen Michael H. (2014). Impact of Age and Gender on the Prevalence and Prognostic Importance of the Metabolic Syndrome and Its Components in Europeans. The MORGAM Prospective Cohort Project. PLoS ONE.

[cit48] Lizcano Fernando, Guzmán Guillermo (2014). Estrogen Deficiency and the Origin of Obesity during Menopause. BioMed Research International.

[cit49] Zhu Jing, Zhou Yier, Jin Bihui, Shu Jing (2023). Role of estrogen in the regulation of central and peripheral energy homeostasis: from a menopausal perspective. Therapeutic Advances in Endocrinology and Metabolism.

[cit50] Drapkina O. M., Kontsevaya A. V., Kalinina A. M., Avdeev S. M., Agaltsov M. V., Alexandrova L. M., Antsiferova A. A., Aronov D. M., Akhmedzhanov N. M., Balanova Yu. A., Balakhonova T. V., Berns S. A., Bochkarev M. V., Bochkareva E. V., Bubnova M. V., Budnevsky A. V., Gambaryan M. G., Gorbunov V. M., Gorny B. E., Gorshkov A. Yu., Gumanova N. G., Dadaeva V. A., Drozdova L. Yu., Egorov V. A., Eliashevich S. O., Ershova A. I., Ivanova E. S., Imaeva A. E., Ipatov P. V., Kaprin A. D., Karamnova N. S., Kobalava Zh. D., Konradi A. O., Kopylova O. V., Korostovtseva L. S., Kotova M. B., Kulikova M. S., Lavrenova E. A., Lischenko O. V., Lopatina M. V., Lukina Yu. V., Lukyanov M. M., Mayev I. V., Mamedov M. N., Markelova S. V., Martsevich S. Yu., Metelskaya V. A., Meshkov A. N., Milushkina O. Yu., Mukaneeva D. K., Myrzamatova A. O., Nebieridze D. V., Orlov D. O., Poddubskaya E. A., Popovich M. V., Popovkina O. E., Potievskaya V. I., Prozorova G. G., Rakovskaya Yu. S., Rotar O. P., Rybakov I. A., Sviryaev Yu. V., Skripnikova I. A., Skoblina N. A., Smirnova M. I., Starinsky V. V., Tolpygina S. N., Usova E. V., Khailova Zh. V., Shalnova S. A., Shepel R. N., Shishkova V. N., Yavelov I. S. (2022). 2022 Prevention of chronic non-communicable diseases in Of the Russian Federation. National guidelines. Cardiovascular Therapy and Prevention.

[cit51] Yureneva Yureneva S.V. (2024). Obesity during the menopausal transition: how to break negative connections and prevent consequences?. Akusherstvo i ginekologiia.

[cit52] Chen Guo-Chong, Arthur Rhonda, Iyengar Neil M, Kamensky Victor, Xue Xiaonan, Wassertheil-Smoller Sylvia, Allison Matthew A, Shadyab Aladdin H, Wild Robert A, Sun Yangbo, Banack Hailey R, Chai Jin Choul, Wactawski-Wende Jean, Manson JoAnn E, Stefanick Marcia L, Dannenberg Andrew J, Rohan Thomas E, Qi Qibin (2019). Association between regional body fat and cardiovascular disease risk among postmenopausal women with normal body mass index. European Heart Journal.

[cit53] Ministry of Health of Russian Federation. Clinical recommendations. Obesity. 2024. Av. at: https://cr.minzdrav.gov.ru/preview-cr/28_3]. [Russian: Ministerstvo zdravookhraneniya Rossiiskoi Federatsii. Klinicheskie rekomendatsii. Ozhirenie. 2024. Dostupno na: https://cr.minzdrav.gov.ru/preview-cr/28_3]

[cit54] Ministerstvo zdravookhraneniya Rossiiskoi Federatsii. Klinicheskie rekomendatsii. Arterial'naya gipertenziya u vzroslykh. 2024. Dostupno na: https://cr.minzdrav.gov.ru/preview-cr/62_3

[cit55] Alberti K George MM, Zimmet Paul, Shaw Jonathan (2005). The metabolic syndrome—a new worldwide definition. The Lancet.

[cit56] Wang Juan, Wu Daichao, Guo Hui, Li Meixiang (2019). Hyperandrogenemia and insulin resistance: The chief culprit of polycystic ovary syndrome. Life Sciences.

[cit57] Cushman Mary (2004). Estrogen Plus Progestin and Risk of Venous Thrombosis. JAMA.

[cit58] Troshina E.A., Pokusaeva V.N., Andreeva E.N. Ozhirenie u zhenshchin. pod red. Mel'nichenko G.A., Nikiforovskogo N.K.- M.: OOO Meditsinskoe informatsionnoe agentstvo; 2017. – 272s ISBN 978-5-9986-0296-2

[cit59] Caprio Massimiliano, Antelmi Antonella, Chetrite Gérard, Muscat Adeline, Mammi Caterina, Marzolla Vincenzo, Fabbri Andrea, Zennaro Maria-Christina, Fève Bruno (2010). Antiadipogenic Effects of the Mineralocorticoid Receptor Antagonist Drospirenone: Potential Implications for the Treatment of Metabolic Syndrome. Endocrinology.

[cit60] Smetnik Smetnik A.A., Ivanov Ivanov I.A., Ermakova Ermakova E.I., Tabeeva Tabeeva G.I. (2025). Characteristics of menopausal hormone therapy use in Russia: results of a large-scale survey of peri- and postmenopausal women. Akusherstvo i ginekologiia.

[cit61] Sukhikh G.T., Serov V.N., Shlyakhto E.V., Dedov I.I., Arutyunov G.P., Tkacheva O.N. i dr. Menopauzal'naya gormonal'naya terapiya u patsientok s serdechno-sosudistymi i metabolicheskimi zabolevaniyami: mezhdistsiplinarnyi del'fiiskii konsensus sredi rossiiskikh ginekologov, kardiologov, endokrinologov, gerontologov i geriatrov, flebologov, klinicheskikh farmakologov. Terapevticheskii arkhiv. 2026;98(1):6–27. doi: https://doi.org/10.26442/00403660.2026.01.203470 41715967

[cit62] Dedov I. I., Shestakova M. V., Vikulova O. K., Zheleznyakova A. V., Isakov M. А. (2021). Epidemiological characteristics of diabetes mellitus in the Russian Federation: clinical and statistical analysis according to the Federal diabetes register data of 01.01.2021. Diabetes mellitus.

[cit63] Salpeter S. R., Walsh J. M. E., Ormiston T. M., Greyber E., Buckley N. S., Salpeter E. E. (2005). Meta‐analysis: effect of hormone‐replacement therapy on components of the metabolic syndrome in postmenopausal women. Diabetes, Obesity and Metabolism.

[cit64] Schindler Adolf E., Campagnoli Carlo, Druckmann René, Huber Johannes, Pasqualini Jorge R., Schweppe Karl W., Thijssen Jos H.H. (2008). Reprint of Classification and pharmacology of progestins. Maturitas.

[cit65] Mendoza Nicolás, Ramírez Isabel, de la Viuda Esther, Coronado Pluvio, Baquedano Laura, Llaneza Plácido, Nieto Verónica, Otero Borja, Sánchez-Méndez Sonia, de Frutos Visitación Álvarez, Andraca Leire, Barriga Patricio, Benítez Zully, Bombas Teresa, Cancelo Mª. Jesús, Cano Antonio, Branco Camil Castelo, Correa Marta, Doval José Luis, Fasero María, Fiol Gabriel, Garello Nestor C., Genazzani Andrea R., Gómez Ana Isabel, Gómez Mª. Ángeles, González Silvia, Goulis Dimitrios G., Guinot Misericordia, Hernández Luis Rolando, Herrero Sonia, Iglesias Eva, Jurado Ana Rosa, Lete Iñaki, Lubián Daniel, Martínez Milagros, Nieto Aníbal, Nieto Laura, Palacios Santiago, Pedreira Milagros, Pérez-Campos Ezequiel, Plá María Jesús, Presa Jesús, Quereda Francisco, Ribes Miriam, Romero Pablo, Roca Beatriz, Sánchez-Capilla Antonio, Sánchez-Borrego Rafael, Santaballa Ana, Santamaría Amparo, Simoncini Tommaso, Tinahones Francisco, Calaf Joaquín (2022). Eligibility criteria for Menopausal Hormone Therapy (MHT): a position statement from a consortium of scientific societies for the use of MHT in women with medical conditions. MHT Eligibility Criteria Group. Maturitas.

[cit66] De Filippis Anna, Ullah Hammad, Baldi Alessandra, Dacrema Marco, Esposito Cristina, Garzarella Emanuele Ugo, Santarcangelo Cristina, Tantipongpiradet Ariyawan, Daglia Maria (2020). Gastrointestinal Disorders and Metabolic Syndrome: Dysbiosis as a Key Link and Common Bioactive Dietary Components Useful for their Treatment. International Journal of Molecular Sciences.

[cit67] Zuvarox T, Goosenberg E, Belletieri C. Malabsorption Syndromes. In: StatPearls-Treasure Island (FL): StatPearls Publishing; 2025. PMID: 31971746 31971746

[cit68] Elkafas Hoda, Walls Melinique, Al-Hendy Ayman, Ismail Nahed (2022). Gut and genital tract microbiomes: Dysbiosis and link to gynecological disorders. Frontiers in Cellular and Infection Microbiology.

[cit69] Xie Xinyuan, Song Jinbin, Wu Yue, Li Mei, Guo Wenfeng, Li Shuang, Li Yanwu (2024). Study on gut microbiota and metabolomics in postmenopausal women. BMC Women's Health.

[cit70] (2022). The 2022 hormone therapy position statement of The North American Menopause Society. Menopause.

[cit71] Kim Ji-Eun, Chang Jae-Hyuck, Jeong Min-Ji, Choi Jaesung, Park JooYong, Baek Chaewon, Shin Aesun, Park Sang Min, Kang Daehee, Choi Ji-Yeob (2020). A systematic review and meta-analysis of effects of menopausal hormone therapy on cardiovascular diseases. Scientific Reports.

[cit72] Boardman Henry MP, Hartley Louise, Eisinga Anne, Main Caroline, Roqué i Figuls Marta, Bonfill Cosp Xavier, Gabriel Sanchez Rafael, Knight Beatrice (2015). Hormone therapy for preventing cardiovascular disease in post-menopausal women. Cochrane Database of Systematic Reviews.

[cit73] Vinogradova Yana, Coupland Carol, Hippisley-Cox Julia (2019). Use of hormone replacement therapy and risk of venous thromboembolism: nested case-control studies using the QResearch and CPRD databases. BMJ.

[cit74] Goldštajn Marina Šprem, Mikuš Mislav, Ferrari Filippo Alberto, Bosco Mariachiara, Uccella Stefano, Noventa Marco, Török Peter, Terzic Sanja, Laganà Antonio Simone, Garzon Simone (2022). Effects of transdermal versus oral hormone replacement therapy in postmenopause: a systematic review. Archives of Gynecology and Obstetrics.

[cit75] Kapoor Ekta, Kling Juliana M., Lobo Angie S., Faubion Stephanie S. (2021). Menopausal hormone therapy in women with medical conditions. Best Practice & Research Clinical Endocrinology & Metabolism.

[cit76] Morris Guy, Talaulikar Vikram (2022). Hormone replacement therapy in women with history of thrombosis or a thrombophilia. Post Reproductive Health.

[cit77] Dinger J., Bardenheuer K., Heinemann K. (2016). Drospirenone plus estradiol and the risk of serious cardiovascular events in postmenopausal women. Climacteric.

[cit78] Sobel Talia H., Shen Wen (2022). Transdermal estrogen therapy in menopausal women at increased risk for thrombotic events: a scoping review. Menopause.

[cit79] Roetker Nicholas, MacLehose Richard, Hoogeveen Ron, Ballantyne Christie, Basu Saonli, Cushman Mary, Folsom Aaron (2018). Prospective Study of Endogenous Hormones and Incidence of Venous Thromboembolism: The Atherosclerosis Risk in Communities Study. Thrombosis and Haemostasis.

[cit80] Tepper Naomi K., Whiteman Maura K., Marchbanks Polly A., James Andra H., Curtis Kathryn M. (2016). Progestin-only contraception and thromboembolism: A systematic review. Contraception.

[cit81] Mantha S., Karp R., Raghavan V., Terrin N., Bauer K. A., Zwicker J. I. (2012). Assessing the risk of venous thromboembolic events in women taking progestin-only contraception: a meta-analysis. BMJ.

[cit82] Nudy Matthew, Chinchilli Vernon M., Foy Andrew J. (2019). A systematic review and meta-regression analysis to examine the ‘timing hypothesis’ of hormone replacement therapy on mortality, coronary heart disease, and stroke. IJC Heart & Vasculature.

[cit83] Cho Leslie, Kaunitz Andrew M., Faubion Stephanie S., Hayes Sharonne N., Lau Emily S., Pristera Nicole, Scott Nandita, Shifren Jan L., Shufelt Chrisandra L., Stuenkel Cynthia A., Lindley Kathryn J. (2023). Rethinking Menopausal Hormone Therapy: For Whom, What, When, and How Long?. Circulation.

[cit84] LaVasseur Corinne, Neukam Suvi, Kartika Thomas, Samuelson Bannow Bethany, Shatzel Joseph, DeLoughery Thomas G. (2022). Hormonal therapies and venous thrombosis: Considerations for prevention and management. Research and Practice in Thrombosis and Haemostasis.

[cit85] Roach Rachel E. J., Lijfering Willem M., van Hylckama Vlieg Astrid, Helmerhorst Frans M., Rosendaal Frits R., Cannegieter Suzanne C. (2013). The risk of venous thrombosis in individuals with a history of superficial vein thrombosis and acquired venous thrombotic risk factors. Blood.

[cit86] Straczek Céline, Oger Emmanuel, Yon de Jonage-Canonico Marianne Beau, Plu-Bureau Geneviève, Conard Jacqueline, Meyer Guy, Alhenc-Gelas Martine, Lévesque Hervé, Trillot Nathalie, Barrellier Marie-Thérèse, Wahl Denis, Emmerich Joseph, Scarabin Pierre-Yves (2005). Prothrombotic Mutations, Hormone Therapy, and Venous Thromboembolism Among Postmenopausal Women. Circulation.

[cit87] Middeldorp Saskia, Nieuwlaat Robby, Baumann Kreuziger Lisa, Coppens Michiel, Houghton Damon, James Andra H., Lang Eddy, Moll Stephan, Myers Tarra, Bhatt Meha, Chai-Adisaksopha Chatree, Colunga-Lozano Luis E., Karam Samer G., Zhang Yuan, Wiercioch Wojtek, Schünemann Holger J., Iorio Alfonso (2023). American Society of Hematology 2023 guidelines for management of venous thromboembolism: thrombophilia testing. Blood Advances.

[cit88] Guyatt Gordon H., Akl Elie A., Crowther Mark, Gutterman David D., Schuünemann Holger J. (2012). Executive Summary. Chest.

[cit89] Hulley Stephen (2003). Randomized Trial of Estrogen Plus Progestin for Secondary Prevention of Coronary Heart Disease in Postmenopausal Women. JAMA.

[cit90] Yuk Jin-Sung, Kim Gwang Sil, Kim Dong-Gil, Byun Young Sup, Kim Myoung-Hwan, Yoon Sang-Hee, Han Gwan Hee, Kim Byung Gyu (2025). Association of menopausal hormone therapy with risk of cardiovascular disease in Korean women. European Journal of Endocrinology.

[cit91] Wassertheil-Smoller Sylvia, Hendrix Susan, Limacher Marian, Heiss Gerardo, Kooperberg Charles, Baird Alison, Kotchen Theodore, Curb J. David, Black Henry, Rossouw Jacques E., Aragaki Aaron, Safford Monika, Stein Evan, Laowattana Somchai, Mysiw W. Jerry, for the WHI Investigators (2003). Effect of Estrogen Plus Progestin on Stroke in Postmenopausal Women. JAMA.

[cit92] Hendrix Susan L., Wassertheil-Smoller Sylvia, Johnson Karen C., Howard Barbara V., Kooperberg Charles, Rossouw Jacques E., Trevisan Maurizio, Aragaki Aaron, Baird Alison E., Bray Paul F., Buring Julie E., Criqui Michael H., Herrington David, Lynch John K., Rapp Stephen R., Torner James (2006). Effects of Conjugated Equine Estrogen on Stroke in the Women’s Health Initiative. Circulation.

[cit93] te West Nevine I. D., Day Ric O., Hiley Basia, White Chris , Wright Mike, Moore Kate H. (2020). Estriol serum levels in new and chronic users of vaginal estriol cream: A prospective observational study. Neurourology and Urodynamics.

[cit94] Santen Richard J., Mirkin Sebastian, Bernick Brian, Constantine Ginger D. (2019). Systemic estradiol levels with low-dose vaginal estrogens. Menopause.

[cit95] Bhupathiraju Shilpa N., Grodstein Francine, Stampfer Meir J., Willett Walter C., Crandall Carolyn J., Shifren Jan L., Manson JoAnn E. (2018). Vaginal estrogen use and chronic disease risk in the Nurses’ Health Study. Menopause.

[cit96] Crandall Carolyn J., Hovey Kathleen M., Andrews Christopher A., Chlebowski Rowan T., Stefanick Marcia L., Lane Dorothy S., Shifren Jan, Chen Chu, Kaunitz Andrew M., Cauley Jane A., Manson JoAnn E. (2017). Breast cancer, endometrial cancer, and cardiovascular events in participants who used vaginal estrogen in the Women's Health Initiative Observational Study. Menopause.

[cit97] Orlova I.A., Plisyuk A.G., Dolgushin G.O., Kirillova K.I., Mikheev R.K., Andreeva E.N. (2023). Correlation between prolonged menopausal hormonotherapy and indicators of vascular and replicative aging in women. Russian Journal of Preventive Medicine.

[cit98] Draper Michael W., Flowers David E., Huster William J., Neild Julie A., Harper Kristine D., Arnaud Claude (2010). A controlled trial of raloxifene (LY139481) HCl: Impact on bone turnover and serum lipid profile in healthy postmenopausal women. Journal of Bone and Mineral Research.

[cit99] Nie Guangning, Yang Xiaofei, Wang Yangyang, Liang Wanshi, Li Xuewen, Luo Qiyuan, Yang Hongyan, Liu Jian, Wang Jiajing, Guo Qinghua, Yu Qi, Liang Xuefang (2022). The Effects of Menopause Hormone Therapy on Lipid Profile in Postmenopausal Women: A Systematic Review and Meta-Analysis. Frontiers in Pharmacology.

[cit100] Ezhov M. V., Kukharchuk V. V., Sergienko I. V., Alieva A. S., Antsiferov M. B., Ansheles A. A., Arabidze G. G., Aronov D. M., Arutyunov G. P., Akhmedzhanov N. M., Balakhonova T. V., Barbarash O. L., Boytsov S. A., Bubnova M. G., Voevoda M. I., Galstyan G. R., Galyavich A. S., Gornyakova N. B., Gurevich V. S., Dedov I. I., Drapkina O. M., Duplyakov D. V., Eregin S. Ya., Ershova A. I., Irtyuga O. B., Karpov R. S., Karpov Yu. A., Kachkovsky M. A., Kobalava Zh. D., Koziolova N. A., Konovalov G. A., Konstantinov V. O., Kosmacheva E. D., Kotovskaya Yu. V., Martynov A. I., Meshkov A. N., Nebieridze D. V., Nedogoda S. V., Obrezan A. G., Oleinikov V. E., Pokrovsky S. N., Ragino Yu. I., Rotar O. P., Skibitsky V. V., Smolenskaya O. G., Sokolov A. A., Sumarokov A. B., Filippov E., Halimov Yu. Sh., Chazova I. E., Shaposhnik I. I., Shestakova M. V., Yakushin S. S., Shlyakhto E. V. (2023). Disorders of lipid metabolism. Clinical Guidelines 2023. Russian Journal of Cardiology.

[cit101] Nordestgaard Børge G., Chapman M. John, Ray Kausik, Borén Jan, Andreotti Felicita, Watts Gerald F., Ginsberg Henry, Amarenco Pierre, Catapano Alberico, Descamps Olivier S., Fisher Edward, Kovanen Petri T., Kuivenhoven Jan Albert, Lesnik Philippe, Masana Luis, Reiner Zeljko, Taskinen Marja-Riitta, Tokgözoglu Lale, Tybjærg-Hansen Anne (2010). Lipoprotein(a) as a cardiovascular risk factor: current status. European Heart Journal.

[cit102] Honigberg Michael C., Trinder Mark, Natarajan Pradeep (2022). Lipoprotein(a), Menopausal Hormone Therapy, and Risk of Coronary Heart Disease in Postmenopausal Individuals. JAMA Cardiology.

[cit103] Stevenson John C., Chines Arkadi, Pan Kaijie, Ryan Kelly A., Mirkin Sebastian (2015). A Pooled Analysis of the Effects of Conjugated Estrogens/Bazedoxifene on Lipid Parameters in Postmenopausal Women From the Selective Estrogens, Menopause, and Response to Therapy (SMART) Trials. The Journal of Clinical Endocrinology & Metabolism.

[cit104] Miller Valery T. (2011). Effects of Estrogen or Estrogen/ Progestin Regimens on Heart Disease Risk Factors in Postmenopausal Women. JAMA.

[cit105] Binder Ellen F., Birge Stanley J., Kohrt Wendy M. (2015). Effects of Endurance Exercise and Hormone Replacement Therapy on Serum Lipids in Older Women. Journal of the American Geriatrics Society.

[cit106] Bunyavejchevin S, Limpaphayom KK. The metabolic and bone density effects of continuous combined 17-beta estradiol and noresthisterone acetate treatments in Thai postmenopausal women: a doubleblind placebo-controlled trial. Journal of the Medical Association of Thailand. 2001;84(1):45–53. PMID: 11281499 11281499

[cit107] Çayan F, Gen R, Akbay E, Dilek U, Dilek S. The Effect of Hormone Therapy and Tibolone on Glucose and Lipid Metabolism in Healthy Postmenopausal Women. Turkish Journal of Geriatrics. 2011;14(1):19– 25. [Av. at: https://geriatri.dergisi.org/abstract.php?id=534]

[cit108] Cheng GJ, Liu JL, Zhang Q, Fan W, Ye HF, Wang ZQ et al. Nylestriol replacement therapy in postmenopausal women. A three-year prospective study. Chinese Medical Journal. 1993;106(12):911–6. PMID: 8198628 8198628

[cit109] Conard Jacqueline, Basdevant Arnaud, Thomas Jean-Louis, Ochsenbein Edith, Denis Catherine, Guyene Than Tam, Degrelle Hervé (2016). Cardiovascular risk factors and combined estrogen-progestin replacement therapy: a placebo-controlled study with nomegestrol acetate and estradiol. Fertility and Sterility.

[cit110] Conard Jacqueline, Gompel Anne, Pelissier Clara, Mirabel Catherine, Basdevant Arnaud (2002). Fibrinogen and plasminogen modifications during oral estradiol replacement therapy. Fertility and Sterility.

[cit111] Davidson Michael H., Maki Kevin C., Marx Phyllis, Maki Ann C., Cyrowski Mary Sue, Nanavati Nayan, Arce Joan-Carles (2003). Effects of Continuous Estrogen and Estrogen-Progestin Replacement Regimens on Cardiovascular Risk Markers in Postmenopausal Women. Archives of Internal Medicine.

[cit112] Duvernoy Claire S., Rose Patricia A., Kim H. Myra, Kehrer Christine, Brook Robert D. (2007). Combined Continuous Ethinyl Estradiol/Norethindrone Acetate Does Not Improve Forearm Blood Flow in Postmenopausal Women at Risk for Cardiovascular Events: A Pilot Study. Journal of Women's Health.

[cit113] Casanova G., dos Reis A. M., Spritzer P. M. (2014). Low-dose oral or non-oral hormone therapy: effects on C-reactive protein and atrial natriuretic peptide in menopause. Climacteric.

[cit114] Haase Christiane L., Tybjærg-Hansen Anne, Ali Qayyum Abbas, Schou Jesper, Nordestgaard Børge G., Frikke-Schmidt Ruth (2011). LCAT, HDL Cholesterol and Ischemic Cardiovascular Disease: A Mendelian Randomization Study of HDL Cholesterol in 54,500 Individuals. The Journal of Clinical Endocrinology & Metabolism.

[cit115] Lv Changyu, Zhang Wencui, Tan Xia, Shang Xianping, Găman Mihnea-Alexandru, Salem Hany, Abu-Zaid Ahmed, Wang Xiaohong (2021). The effect of tibolone treatment on lipid profile in women: A systematic review and dose-response meta-analysis of randomized controlled trials. Pharmacological Research.

[cit116] Anagnostis Panagiotis, Galanis Petros, Chatzistergiou Vasileia, Stevenson John C., Godsland Ian F., Lambrinoudaki Irene, Theodorou Mamas, Goulis Dimitrios G. (2017). The effect of hormone replacement therapy and tibolone on lipoprotein (a) concentrations in postmenopausal women: A systematic review and meta-analysis. Maturitas.

[cit117] FALKEBORN MARGARETA, PERSSON INGEMAR, ADAMI HANS‐OLOV, BERGSTRÖM REINHOLD, EAKER ELAINE, LITHELL HANS, MOHSEN RAWYA, NAESSEN TORD (2005). The risk of acute myocardial infarction after oestrogen and oestrogen‐progestogen replacement. BJOG: An International Journal of Obstetrics & Gynaecology.

[cit118] Shufelt Chrisandra L, Manson JoAnn E (2021). Menopausal Hormone Therapy and Cardiovascular Disease: The Role of Formulation, Dose, and Route of Delivery. The Journal of Clinical Endocrinology & Metabolism.

[cit119] Anagnostis Panagiotis, Bitzer Johannes, Cano Antonio, Ceausu Iuliana, Chedraui Peter, Durmusoglu Fatih, Erkkola Risto, Goulis Dimitrios G., Hirschberg Angelica Lindén, Kiesel Ludwig, Lopes Patrice, Pines Amos, van Trotsenburg Mick, Lambrinoudaki Irene, Rees Margaret (2020). Menopause symptom management in women with dyslipidemias: An EMAS clinical guide. Maturitas.

[cit120] Wenger Nanette K., Lloyd-Jones Donald M., Elkind Mitchell S.V., Fonarow Gregg C., Warner John J., Alger Heather M., Cheng Susan, Kinzy Claire, Hall Jennifer L., Roger Véronique L. (2022). Call to Action for Cardiovascular Disease in Women: Epidemiology, Awareness, Access, and Delivery of Equitable Health Care: A Presidential Advisory From the American Heart Association. Circulation.

[cit121] Vogel Birgit, Acevedo Monica, Appelman Yolande, Bairey Merz C Noel, Chieffo Alaide, Figtree Gemma A, Guerrero Mayra, Kunadian Vijay, Lam Carolyn S P, Maas Angela H E M, Mihailidou Anastasia S, Olszanecka Agnieszka, Poole Jeanne E, Saldarriaga Clara, Saw Jacqueline, Zühlke Liesl, Mehran Roxana (2021). The Lancet women and cardiovascular disease Commission: reducing the global burden by 2030. The Lancet.

[cit122] Reckelhoff Jane F. (2018). Gender differences in hypertension. Current Opinion in Nephrology and Hypertension.

[cit123] Cho Leslie, Davis Melinda, Elgendy Islam, Epps Kelly, Lindley Kathryn J., Mehta Puja K., Michos Erin D., Minissian Margo, Pepine Carl, Vaccarino Viola, Volgman Annabelle Santos (2020). Summary of Updated Recommendations for Primary Prevention of Cardiovascular Disease in Women. Journal of the American College of Cardiology.

[cit124] Gerdts Eva, Sudano Isabella, Brouwers Sofie, Borghi Claudio, Bruno Rosa Maria, Ceconi Claudio, Cornelissen Véronique, Diévart François, Ferrini Marc, Kahan Thomas, Løchen Maja-Lisa, Maas Angela H E M, Mahfoud Felix, Mihailidou Anastasia S, Moholdt Trine, Parati Gianfranco, de Simone Giovanni (2022). Sex differences in arterial hypertension. European Heart Journal.

[cit125] Zhou Bin, Carrillo-Larco Rodrigo M, Danaei Goodarz, Riley Leanne M, Paciorek Christopher J, Stevens Gretchen A, Gregg Edward W, Bennett James E, Solomon Bethlehem, Singleton Rosie K, Sophiea Marisa K, Iurilli Maria LC, Lhoste Victor PF, Cowan Melanie J, Savin Stefan, Woodward Mark, Balanova Yulia, Cifkova Renata, Damasceno Albertino, Elliott Paul, Farzadfar Farshad, He Jiang, Ikeda Nayu, Kengne Andre P, Khang Young-Ho, Kim Hyeon Chang, Laxmaiah Avula, Lin Hsien-Ho, Margozzini Maira Paula, Miranda J Jaime, Neuhauser Hannelore, Sundström Johan, Varghese Cherian, Widyahening Indah S, Zdrojewski Tomasz, Abarca-Gómez Leandra, Abdeen Ziad A, Abdul Rahim Hanan F, Abu-Rmeileh Niveen M, Acosta-Cazares Benjamin, Adams Robert J, Aekplakorn Wichai, Afsana Kaosar, Afzal Shoaib, Agdeppa Imelda A, Aghazadeh-Attari Javad, Aguilar-Salinas Carlos A, Agyemang Charles, Ahmad Noor Ani, Ahmadi Ali, Ahmadi Naser, Ahmadi Nastaran, Ahmadizar Fariba, Ahmed Soheir H, Ahrens Wolfgang, Ajlouni Kamel, Al-Raddadi Rajaa, Alarouj Monira, AlBuhairan Fadia, AlDhukair Shahla, Ali Mohamed M, Alkandari Abdullah, Alkerwi Ala'a, Allin Kristine, Aly Eman, Amarapurkar Deepak N, Amougou Norbert, Amouyel Philippe, Andersen Lars Bo, Anderssen Sigmund A, Anjana Ranjit Mohan, Ansari-Moghaddam Alireza, Ansong Daniel, Aounallah-Skhiri Hajer, Araújo Joana, Ariansen Inger, Aris Tahir, Arku Raphael E, Arlappa Nimmathota, Aryal Krishna K, Aspelund Thor, Assah Felix K, Assunção Maria Cecília F, Auvinen Juha, Avdićová Mária, Azevedo Ana, Azimi-Nezhad Mohsen, Azizi Fereidoun, Azmin Mehrdad, Babu Bontha V, Bahijri Suhad, Balakrishna Nagalla, Bamoshmoosh Mohamed, Banach Maciej, Banadinović Maja, Bandosz Piotr, Banegas José R, Baran Joanna, Barbagallo Carlo M, Barceló Alberto, Barkat Amina, Barreto Marta, Barros Aluisio JD, Barros Mauro Virgílio Gomes, Bartosiewicz Anna, Basit Abdul, Bastos Joao Luiz D, Bata Iqbal, Batieha Anwar M, Batyrbek Assembekov, Baur Louise A, Beaglehole Robert, Belavendra Antonisamy, Ben Romdhane Habiba, Benet Mikhail, Benson Lowell S, Berkinbayev Salim, Bernabe-Ortiz Antonio, Bernotiene Gailute, Bettiol Heloísa, Bezerra Jorge, Bhagyalaxmi Aroor, Bhargava Santosh K, Bia Daniel, Biasch Katia, Bika Lele Elysée Claude, Bikbov Mukharram M, Bista Bihungum, Bjerregaard Peter, Bjertness Espen, Bjertness Marius B, Björkelund Cecilia, Bloch Katia V, Blokstra Anneke, Bo Simona, Bobak Martin, Boeing Heiner, Boggia Jose G, Boissonnet Carlos P, Bojesen Stig E, Bongard Vanina, Bonilla-Vargas Alice, Bopp Matthias, Borghs Herman, Bovet Pascal, Boyer Christopher B, Braeckman Lutgart, Brajkovich Imperia, Branca Francesco, Breckenkamp Juergen, Brenner Hermann, Brewster Lizzy M, Briceño Yajaira, Brito Miguel, Bruno Graziella, Bueno-de-Mesquita H Bas, Bueno Gloria, Bugge Anna, Burns Con, Bursztyn Michael, Cabrera de León Antonio, Cacciottolo Joseph, Cameron Christine, Can Günay, Cândido Ana Paula C, Capanzana Mario V, Čapková Naděžda, Capuano Eduardo, Capuano Vincenzo, Cardoso Viviane C, Carlsson Axel C, Carvalho Joana, Casanueva Felipe F, Censi Laura, Cervantes-Loaiza Marvin, Chadjigeorgiou Charalambos A, Chamukuttan Snehalatha, Chan Angelique W, Chan Queenie, Chaturvedi Himanshu K, Chaturvedi Nish, Chee Miao Li, Chen Chien-Jen, Chen Fangfang, Chen Huashuai, Chen Shuohua, Chen Zhengming, Cheng Ching-Yu, Cheraghian Bahman, Cherkaoui Dekkaki Imane, Chetrit Angela, Chien Kuo-Liong, Chiolero Arnaud, Chiou Shu-Ti, Chirita-Emandi Adela, Chirlaque María-Dolores, Cho Belong, Christensen Kaare, Christofaro Diego G, Chudek Jerzy, Cinteza Eliza, Claessens Frank, Clarke Janine, Clays Els, Cohen Emmanuel, Concin Hans, Cooper Cyrus, Coppinger Tara C, Costanzo Simona, Cottel Dominique, Cowell Chris, Craig Cora L, Crampin Amelia C, Crujeiras Ana B, Cruz Juan J, Csilla Semánová, Cui Liufu, Cureau Felipe V, Cuschieri Sarah, D'Arrigo Graziella, d'Orsi Eleonora, Dallongeville Jean, Dankner Rachel, Dantoft Thomas M, Dauchet Luc, Davletov Kairat, De Backer Guy, De Bacquer Dirk, De Curtis Amalia, de Gaetano Giovanni, De Henauw Stefaan, de Oliveira Paula Duarte, De Ridder David, De Smedt Delphine, Deepa Mohan, Deev Alexander D, DeGennaro Vincent Jr, Delisle Hélène, Demarest Stefaan, Dennison Elaine, Deschamps Valérie, Dhimal Meghnath, Di Castelnuovo Augusto F, Dias-da-Costa Juvenal Soares, Diaz Alejandro, Dickerson Ty T, Dika Zivka, Djalalinia Shirin, Do Ha TP, Dobson Annette J, Donfrancesco Chiara, Donoso Silvana P, Döring Angela, Dorobantu Maria, Dörr Marcus, Doua Kouamelan, Dragano Nico, Drygas Wojciech, Duante Charmaine A, Duboz Priscilla, Duda Rosemary B, Dulskiene Virginija, Dushpanova Anar, Džakula Aleksandar, Dzerve Vilnis, Dziankowska-Zaborszczyk Elzbieta, Eddie Ricky, Eftekhar Ebrahim, Eggertsen Robert, Eghtesad Sareh, Eiben Gabriele, Ekelund Ulf, El-Khateeb Mohammad, El Ati Jalila, Eldemire-Shearer Denise, Eliasen Marie, Elosua Roberto, Erasmus Rajiv T, Erbel Raimund, Erem Cihangir, Eriksen Louise, Eriksson Johan G, Escobedo-de la Peña Jorge, Eslami Saeid, Esmaeili Ali, Evans Alun, Faeh David, Fakhretdinova Albina A, Fall Caroline H, Faramarzi Elnaz, Farjam Mojtaba, Fattahi Mohammad Reza, Fawwad Asher, Felix-Redondo Francisco J, Felix Stephan B, Ferguson Trevor S, Fernandes Romulo A, Fernández-Bergés Daniel, Ferrante Daniel, Ferrao Thomas, Ferrari Marika, Ferrario Marco M, Ferreccio Catterina, Ferreira Haroldo S, Ferrer Eldridge, Ferrieres Jean, Figueiró Thamara Hubler, Fink Günther, Fischer Krista, Foo Leng Huat, Forsner Maria, Fouad Heba M, Francis Damian K, Franco Maria do Carmo, Frikke-Schmidt Ruth, Frontera Guillermo, Fuchs Flavio D, Fuchs Sandra C, Fujita Yuki, Fumihiko Matsuda, Furdela Viktoriya, Furer Ariel, Furusawa Takuro, Gaciong Zbigniew, Galbarczyk Andrzej, Galenkamp Henrike, Galvano Fabio, Gao Jingli, Gao Pei, Garcia-de-la-Hera Manoli, Garcia Pablo, Gareta Dickman, Garnett Sarah P, Gaspoz Jean-Michel, Gasull Magda, Gazzinelli Andrea, Gehring Ulrike, Geleijnse Johanna M, George Ronnie, Ghanbari Ali, Ghasemi Erfan, Gheorghe-Fronea Oana-Florentina, Ghimire Anup, Gialluisi Alessandro, Giampaoli Simona, Gieger Christian, Gill Tiffany K, Giovannelli Jonathan, Gironella Glen, Giwercman Aleksander, Gkiouras Konstantinos, Goldberg Marcel, Goldsmith Rebecca A, Gomez Luis F, Gomula Aleksandra, Gonçalves Helen, Gonçalves Mauer, Gonçalves Cordeiro da Silva Bruna, Gonzalez-Chica David A, Gonzalez-Gross Marcela, González-Rivas Juan P, González-Villalpando Clicerio, González-Villalpando María-Elena, Gonzalez Angel R, Gorbea Mariano Bonet, Gottrand Frederic, Graff-Iversen Sidsel, Grafnetter Dušan, Grajda Aneta, Grammatikopoulou Maria G, Gregor Ronald D, Grodzicki Tomasz, Grosso Giuseppe, Gruden Gabriella, Gu Dongfeng, Guan Ong Peng, Gudmundsson Elias F, Gudnason Vilmundur, Guerrero Ramiro, Guessous Idris, Guimaraes Andre L, Gulliford Martin C, Gunnlaugsdottir Johanna, Gunter Marc J, Gupta Prakash C, Gupta Rajeev, Gureje Oye, Gurzkowska Beata, Gutierrez Laura, Gutzwiller Felix, Ha Seongjun, Hadaegh Farzad, Haghshenas Rosa, Hakimi Hamid, Halkjær Jytte, Hambleton Ian R, Hamzeh Behrooz, Hange Dominique, Hanif Abu AM, Hantunen Sari, Hao Jie, Hardman Carla Menêses, Hari Kumar Rachakulla, Hashemi-Shahri Seyed Mohammad, Hata Jun, Haugsgjerd Teresa, Hayes Alison J, He Yuna, Heier Margit, Hendriks Marleen Elisabeth, Henrique Rafael dos Santos, Henriques Ana, Hernandez Cadena Leticia, Herqutanto, Herrala Sauli, Heshmat Ramin, Hill Allan G, Ho Sai Yin, Ho Suzanne C, Hobbs Michael, Holdsworth Michelle, Homayounfar Reza, Horasan Dinc Gonul, Horimoto Andrea RVR, Hormiga Claudia M, Horta Bernardo L, Houti Leila, Howitt Christina, Htay Thein Thein, Htet Aung Soe, Htike Maung Maung Than, Hu Yonghua, Huerta José María, Huhtaniemi Ilpo Tapani, Huiart Laetitia, Huisman Martijn, Husseini Abdullatif S, Huybrechts Inge, Hwalla Nahla, Iacoviello Licia, Iannone Anna G, Ibrahim Mohsen M, Ibrahim Wong Norazizah, Ikram M Arfan, Iotova Violeta, Irazola Vilma E, Ishida Takafumi, Isiguzo Godsent C, Islam Muhammad, Islam Sheikh Mohammed Shariful, Iwasaki Masanori, Jackson Rod T, Jacobs Jeremy M, Jaddou Hashem Y, Jafar Tazeen, James Kenneth, Jamrozik Konrad, Janszky Imre, Janus Edward, Jarvelin Marjo-Riitta, Jasienska Grazyna, Jelaković Ana, Jelaković Bojan, Jennings Garry, Jha Anjani Kumar, Jiang Chao Qiang, Jimenez Ramon O, Jöckel Karl-Heinz, Joffres Michel, Johansson Mattias, Jokelainen Jari J, Jonas Jost B, Jørgensen Torben, Joshi Pradeep, Joukar Farahnaz, Jóżwiak Jacek, Juolevi Anne, Jurak Gregor, Jureša Vesna, Kaaks Rudolf, Kafatos Anthony, Kajantie Eero O, Kalmatayeva Zhanna, Kalpourtzi Natasa, Kalter-Leibovici Ofra, Kampmann Freja B, Kannan Srinivasan, Karaglani Eva, Kårhus Line L, Karki Khem B, Katibeh Marzieh, Katz Joanne, Kauhanen Jussi, Kaur Prabhdeep, Kavousi Maryam, Kazakbaeva Gyulli M, Keil Ulrich, Keinan Boker Lital, Keinänen-Kiukaanniemi Sirkka, Kelishadi Roya, Kemper Han CG, Keramati Maryam, Kerimkulova Alina, Kersting Mathilde, Key Timothy, Khader Yousef Saleh, Khalili Davood, Khaw Kay-Tee, Kheiri Bahareh, Kheradmand Motahareh, Khosravi Alireza, Kiechl-Kohlendorfer Ursula, Kiechl Stefan, Killewo Japhet, Kim Dong Wook, Kim Jeongseon, Klakk Heidi, Klimek Magdalena, Klumbiene Jurate, Knoflach Michael, Kolle Elin, Kolsteren Patrick, Kontto Jukka P, Korpelainen Raija, Korrovits Paul, Kos Jelena, Koskinen Seppo, Kouda Katsuyasu, Kowlessur Sudhir, Koziel Slawomir, Kratenova Jana, Kriaucioniene Vilma, Kristensen Peter Lund, Krokstad Steiner, Kromhout Daan, Kruger Herculina S, Kubinova Ruzena, Kuciene Renata, Kujala Urho M, Kulaga Zbigniew, Kumar R Krishna, Kurjata Pawel, Kusuma Yadlapalli S, Kutsenko Vladimir, Kuulasmaa Kari, Kyobutungi Catherine, Laatikainen Tiina, Lachat Carl, Laid Youcef, Lam Tai Hing, Landrove Orlando, Lanska Vera, Lappas Georg, Larijani Bagher, Latt Tint Swe, Le Coroller Gwenaëlle, Le Nguyen Bao Khanh, Le Tuyen D, Lee Jeannette, Lee Jeonghee, Lehmann Nils, Lehtimäki Terho, Lemogoum Daniel, Levitt Naomi S, Li Yanping, Lilly Christa L, Lim Wei-Yen, Lima-Costa M Fernanda, Lin Xu, Lin Yi-Ting, Lind Lars, Lingam Vijaya, Linneberg Allan, Lissner Lauren, Litwin Mieczyslaw, Lo Wei-Cheng, Loit Helle-Mai, Lopez-Garcia Esther, Lopez Tania, Lotufo Paulo A, Lozano José Eugenio, Lukačević Lovrenčić Iva, Lukrafka Janice L, Luksiene Dalia, Lundqvist Annamari, Lundqvist Robert, Lunet Nuno, Lustigová Michala, Luszczki Edyta, Ma Guansheng, Ma Jun, Machado-Coelho George LL, Machado-Rodrigues Aristides M, Macia Enguerran, Macieira Luisa M, Madar Ahmed A, Maggi Stefania, Magliano Dianna J, Magriplis Emmanuella, Mahasampath Gowri, Maire Bernard, Majer Marjeta, Makdisse Marcia, Malekzadeh Fatemeh, Malekzadeh Reza, Malhotra Rahul, Mallikharjuna Rao Kodavanti, Malyutina Sofia K, Maniego Lynell V, Manios Yannis, Mann Jim I, Mansour-Ghanaei Fariborz, Manzato Enzo, Marcil Anie, Mårild Staffan B, Marinović Glavić Mihalea, Marques-Vidal Pedro, Marques Larissa Pruner, Marrugat Jaume, Martorell Reynaldo, Mascarenhas Luis P, Matasin Marija, Mathiesen Ellisiv B, Mathur Prashant, Matijasevich Alicia, Matlosz Piotr, Matsha Tandi E, Mavrogianni Christina, Mbanya Jean Claude N, Mc Donald Posso Anselmo J, McFarlane Shelly R, McGarvey Stephen T, McLachlan Stela, McLean Rachael M, McLean Scott B, McNulty Breige A, Mediene Benchekor Sounnia, Medzioniene Jurate, Mehdipour Parinaz, Mehlig Kirsten, Mehrparvar Amir Houshang, Meirhaeghe Aline, Meisinger Christa, Mendoza Montano Carlos, Menezes Ana Maria B, Menon Geetha R, Mereke Alibek, Meshram Indrapal I, Metspalu Andres, Meyer Haakon E, Mi Jie, Michels Nathalie, Mikkel Kairit, Milkowska Karolina, Miller Jody C, Minderico Cláudia S, Mini GK, Mirjalili Mohammad Reza, Mirrakhimov Erkin, Mišigoj-Duraković Marjeta, Modesti Pietro A, Moghaddam Sahar Saeedi, Mohajer Bahram, Mohamed Mostafa K, Mohamed Shukri F, Mohammad Kazem, Mohammadi Mohammad Reza, Mohammadi Zahra, Mohammadifard Noushin, Mohammadpourhodki Reza, Mohan Viswanathan, Mohanna Salim, Mohd Yusoff Muhammad Fadhli, Mohebbi Iraj, Mohebi Farnam, Moitry Marie, Møllehave Line T, Molnár Dénes, Momenan Amirabbas, Mondo Charles K, Monterrubio-Flores Eric, Monyeki Kotsedi Daniel K, Moon Jin Soo, Moosazadeh Mahmood, Moreira Leila B, Morejon Alain, Moreno Luis A, Morgan Karen, Moschonis George, Mossakowska Malgorzata, Mostafa Aya, Mostafavi Seyed-Ali, Mota Jorge, Motlagh Mohammad Esmaeel, Motta Jorge, Moura-dos-Santos Marcos André, Mridha Malay K, Msyamboza Kelias P, Mu Thet Thet, Muhihi Alfa J, Muiesan Maria L, Müller-Nurasyid Martina, Murphy Neil, Mursu Jaakko, Musa Kamarul Imran, Musić Milanović Sanja, Musil Vera, Mustafa Norlaila, Nabipour Iraj, Naderimagham Shohreh, Nagel Gabriele, Naidu Balkish M, Najafi Farid, Nakamura Harunobu, Námešná Jana, Nang Ei Ei K, Nangia Vinay B, Narake Sameer, Ndiaye Ndeye Coumba, Neal William A, Nejatizadeh Azim, Nenko Ilona, Neovius Martin, Nguyen Chung T, Nguyen Nguyen D, Nguyen Quang V, Nguyen Quang Ngoc, Nieto-Martínez Ramfis E, Niiranen Teemu J, Nikitin Yury P, Ninomiya Toshiharu, Nishtar Sania, Njelekela Marina A, Noale Marianna, Noboa Oscar A, Noorbala Ahmad Ali, Norat Teresa, Nordendahl Maria, Nordestgaard Børge G, Noto Davide, Nowak-Szczepanska Natalia, Nsour Mohannad Al, Nunes Baltazar, O'Neill Terence W, O'Reilly Dermot, Ochimana Caleb, Oda Eiji, Odili Augustine N, Oh Kyungwon, Ohara Kumiko, Ohtsuka Ryutaro, Olié Valérie, Olinto Maria Teresa A, Oliveira Isabel O, Omar Mohd Azahadi, Onat Altan, Ong Sok King, Ono Lariane M, Ordunez Pedro, Ornelas Rui, Ortiz Pedro J, Osmond Clive, Ostojic Sergej M, Ostovar Afshin, Otero Johanna A, Overvad Kim, Owusu-Dabo Ellis, Paccaud Fred Michel, Padez Cristina, Pahomova Elena, Paiva Karina Mary de, Pająk Andrzej, Palli Domenico, Palmieri Luigi, Pan Wen-Harn, Panda-Jonas Songhomitra, Panza Francesco, Paoli Mariela, Papandreou Dimitrios, Park Soon-Woo, Park Suyeon, Parnell Winsome R, Parsaeian Mahboubeh, Pasquet Patrick, Patel Nikhil D, Pavlyshyn Halyna, Pećin Ivan, Pednekar Mangesh S, Pedro João M, Peer Nasheeta, Peixoto Sergio Viana, Peltonen Markku, Pereira Alexandre C, Peres Karen GDA, Peres Marco A, Peters Annette, Petkeviciene Janina, Peykari Niloofar, Pham Son Thai, Pichardo Rafael N, Pigeot Iris, Pikhart Hynek, Pilav Aida, Pilotto Lorenza, Pitakaka Freda, Piwonska Aleksandra, Pizarro Andreia n, Plans-Rubió Pedro, Polašek Ozren, Porta Miquel, Poudyal Anil, Pourfarzi Farhad, Pourshams Akram, Poustchi Hossein, Pradeepa Rajendra, Price Alison J, Price Jacqueline F, Providencia Rui, Puhakka Soile E, Puiu Maria, Punab Margus, Qasrawi Radwan F, Qorbani Mostafa, Queiroz Daniel, Quoc Bao Tran, Radić Ivana, Radisauskas Ricardas, Rahimikazerooni Salar, Rahman Mahfuzar, Raitakari Olli, Raj Manu, Rakhimova Ellina M, Ramachandra Rao Sudha, Ramachandran Ambady, Ramos Elisabete, Rampal Lekhraj, Rampal Sanjay, Rangel Reina Daniel A, Rarra Vayia, Rech Cassiano Ricardo, Redon Josep, Reganit Paul Ferdinand M, Regecová Valéria, Revilla Luis, Rezaianzadeh Abbas, Ribeiro Robespierre, Riboli Elio, Richter Adrian, Rigo Fernando, Rinke de Wit Tobias F, Ritti-Dias Raphael M, Robitaille Cynthia, Rodríguez-Artalejo Fernando, Rodriguez-Perez María del Cristo, Rodríguez-Villamizar Laura A, Roggenbuck Ulla, Rojas-Martinez Rosalba, Romaguera Dora, Romeo Elisabetta L, Rosengren Annika, Roy Joel GR, Rubinstein Adolfo, Ruidavets Jean-Bernard, Ruiz-Betancourt Blanca Sandra, Ruiz-Castell Maria, Rusakova Iuliia A, Russo Paola, Rutkowski Marcin, Sabanayagam Charumathi, Sabbaghi Hamideh, Sachdev Harshpal S, Sadjadi Alireza, Safarpour Ali Reza, Safi Sare, Safiri Saeid, Saidi Olfa, Sakarya Sibel, Saki Nader, Salanave Benoit, Salazar Martinez Eduardo, Salmerón Diego, Salomaa Veikko, Salonen Jukka T, Salvetti Massimo, Sánchez-Abanto Jose, Sans Susana, Santos Diana A, Santos Ina S, Santos Lèlita C, Santos Maria Paula, Santos Rute, Saramies Jouko L, Sardinha Luis B, Sarganas Giselle, Sarrafzadegan Nizal, Sathish Thirunavukkarasu, Saum Kai-Uwe, Savva Savvas, Sawada Norie, Sbaraini Mariana, Scazufca Marcia, Schaan Beatriz D, Schargrodsky Herman, Schipf Sabine, Schmidt Carsten O, Schnohr Peter, Schöttker Ben, Schramm Sara, Schultsz Constance, Schutte Aletta E, Sebert Sylvain, Sein Aye Aye, Sen Abhijit, Senbanjo Idowu O, Sepanlou Sadaf G, Servais Jennifer, Shalnova Svetlana A, Shamah-Levy Teresa, Shamshirgaran Morteza, Shanthirani Coimbatore Subramaniam, Sharafkhah Maryam, Sharma Sanjib K, Shaw Jonathan E, Shayanrad Amaneh, Shayesteh Ali Akbar, Shi Zumin, Shibuya Kenji, Shimizu-Furusawa Hana, Shin Dong Wook, Shirani Majid, Shiri Rahman, Shrestha Namuna, Si-Ramlee Khairil, Siani Alfonso, Siantar Rosalynn, Sibai Abla M, Silva Caroline Ramos de Moura, Silva Diego Augusto Santos, Simon Mary, Simons Judith, Simons Leon A, Sjöström Michael, Slowikowska-Hilczer Jolanta, Slusarczyk Przemyslaw, Smeeth Liam, So Hung-Kwan, Soares Fernanda Cunha, Sobngwi Eugène, Söderberg Stefan, Soemantri Agustinus, Sofat Reecha, Solfrizzi Vincenzo, Somi Mohammad Hossein, Sonestedt Emily, Song Yi, Sørensen Thorkild IA, Sørgjerd Elin P, Sorić Maroje, Sossa Jérome Charles, Soumaré Aïcha, Sparboe-Nilsen Bente, Sparrenberger Karen, Staessen Jan A, Starc Gregor, Stavreski Bill, Steene-Johannessen Jostein, Stehle Peter, Stein Aryeh D, Stergiou George S, Stessman Jochanan, Stieber Jutta, Stöckl Doris, Stocks Tanja, Stokwiszewski Jakub, Stronks Karien, Strufaldi Maria Wany, Suka Machi, Sun Chien-An, Sung Yn-Tz, Suriyawongpaisal Paibul, Sy Rody G, Syddall Holly E, Sylva René Charles, Szklo Moyses, Tai E Shyong, Tammesoo Mari-Liis, Tamosiunas Abdonas, Tan Eng Joo, Tang Xun, Tanser Frank, Tao Yong, Tarawneh Mohammed Rasoul, Tarqui-Mamani Carolina B, Taylor Anne, Taylor Julie, Tebar William R, Tell Grethe S, Tello Tania, Tham Yih Chung, Thankappan KR, Theobald Holger, Theodoridis Xenophon, Thijs Lutgarde, Thinggaard Mikael, Thomas Nihal, Thorand Barbara, Thuesen Betina H, Timmermans Erik J, Tjandrarini Dwi H, Tjonneland Anne, Toft Ulla, Tolonen Hanna K, Tolstrup Janne S, Topbas Murat, Topór-Madry Roman, Tormo María José, Tornaritis Michael J, Torrent Maties, Torres-Collado Laura, Touloumi Giota, Traissac Pierre, Triantafyllou Areti, Trichopoulos Dimitrios, Trichopoulou Antonia, Trinh Oanh TH, Trivedi Atul, Tshepo Lechaba, Tsugane Shoichiro, Tuliakova Azaliia M, Tulloch-Reid Marshall K, Tullu Fikru, Tuomainen Tomi-Pekka, Tuomilehto Jaakko, Turley Maria L, Twig Gilad, Tynelius Per, Tzourio Christophe, Ueda Peter, Ugel Eunice, Ulmer Hanno, Uusitalo Hannu MT, Valdivia Gonzalo, Valvi Damaskini, van Dam Rob M, van den Born Bert-Jan, Van der Heyden Johan, van der Schouw Yvonne T, Van Herck Koen, Van Minh Hoang, Van Schoor Natasja M, van Valkengoed Irene GM, van Zutphen Elisabeth M, Vanderschueren Dirk, Vanuzzo Diego, Varbo Anette, Vasan Senthil K, Vega Tomas, Veidebaum Toomas, Velasquez-Melendez Gustavo, Veronesi Giovanni, Verschuren WM Monique, Verstraeten Roosmarijn, Victora Cesar G, Viet Lucie, Villalpando Salvador, Vineis Paolo, Vioque Jesus, Virtanen Jyrki K, Visvikis-Siest Sophie, Viswanathan Bharathi, Vlasoff Tiina, Vollenweider Peter, Voutilainen Ari, Wade Alisha N, Walton Janette, Wambiya Elvis OA, Wan Bebakar Wan Mohamad, Wan Mohamud Wan Nazaimoon, Wanderley Júnior Rildo de Souza, Wang Ming-Dong, Wang Ningli, Wang Qian, Wang Xiangjun, Wang Ya Xing, Wang Ying-Wei, Wannamethee S Goya, Wareham Nicholas, Wei Wenbin, Weres Aneta, Werner Bo, Whincup Peter H, Widhalm Kurt, Wiecek Andrzej, Wilks Rainford J, Willeit Johann, Willeit Peter, Williams Emmanuel A, Wilsgaard Tom, Wojtyniak Bogdan, Wong-McClure Roy A, Wong Andrew, Wong Tien Yin, Woo Jean, Wu Frederick C, Wu Shouling, Wyszynska Justyna, Xu Haiquan, Xu Liang, Yaacob Nor Azwany, Yan Weili, Yang Ling, Yang Xiaoguang, Yang Yang, Yasuharu Tabara, Ye Xingwang, Yiallouros Panayiotis K, Yoosefi Moein, Yoshihara Akihiro, You San-Lin, Younger-Coleman Novie O, Yusoff Ahmad Faudzi, Zainuddin Ahmad A, Zakavi Seyed Rasoul, Zamani Farhad, Zambon Sabina, Zampelas Antonis, Zapata Maria Elisa, Zaw Ko Ko, Zejglicova Kristyna, Zeljkovic Vrkic Tajana, Zeng Yi, Zhang Luxia, Zhang Zhen-Yu, Zhao Dong, Zhao Ming-Hui, Zhen Shiqi, Zheng Yingfeng, Zholdin Bekbolat, Zhu Dan, Zins Marie, Zitt Emanuel, Zocalo Yanina, Zoghlami Nada, Zuñiga Cisneros Julio, Ezzati Majid (2021). Worldwide trends in hypertension prevalence and progress in treatment and control from 1990 to 2019: a pooled analysis of 1201 population-representative studies with 104 million participants. The Lancet.

[cit126] O'Keeffe Linda M., Simpkin Andrew J., Tilling Kate, Anderson Emma L., Hughes Alun D., Lawlor Debbie A., Fraser Abigail, Howe Laura D. (2018). Sex-specific trajectories of measures of cardiovascular health during childhood and adolescence: A prospective cohort study. Atherosclerosis.

[cit127] Ji Hongwei, Kim Andy, Ebinger Joseph E., Niiranen Teemu J., Claggett Brian L., Bairey Merz C. Noel, Cheng Susan (2020). Sex Differences in Blood Pressure Trajectories Over the Life Course. JAMA Cardiology.

[cit128] Maas Angela H E M, Rosano Giuseppe, Cifkova Renata, Chieffo Alaide, van Dijken Dorenda, Hamoda Haitham, Kunadian Vijay, Laan Ellen, Lambrinoudaki Irene, Maclaran Kate, Panay Nick, Stevenson John C, van Trotsenburg Mick, Collins Peter (2020). Cardiovascular health after menopause transition, pregnancy disorders, and other gynaecologic conditions: a consensus document from European cardiologists, gynaecologists, and endocrinologists. European Heart Journal.

[cit129] El Khoudary Samar R., Aggarwal Brooke, Beckie Theresa M., Hodis Howard N., Johnson Amber E., Langer Robert D., Limacher Marian C., Manson JoAnn E., Stefanick Marcia L., Allison Matthew A. (2020). Menopause Transition and Cardiovascular Disease Risk: Implications for Timing of Early Prevention: A Scientific Statement From the American Heart Association. Circulation.

[cit130] Biglia N., Cagnacci A., Gambacciani M., Lello S., Maffei S., Nappi R. E. (2017). Vasomotor symptoms in menopause: a biomarker of cardiovascular disease risk and other chronic diseases?. Climacteric.

[cit131] Chapman Niamh, Ching Siew M., Konradi Aleksandra O., Nuyt Anne Monique, Khan Taskeen, Twumasi-Ankrah Betty, Cho Eun J., Schutte Aletta E., Touyz Rhian M., Steckelings U. Muscha, Brewster Lizzy M. (2023). Arterial Hypertension in Women: State of the Art and Knowledge Gaps. Hypertension.

[cit132] Coutinho Thais (2014). Arterial Stiffness and Its Clinical Implications in Women. Canadian Journal of Cardiology.

[cit133] Picone Dean S., Kodithuwakku Vimarsha, Mayer Christopher C., Chapman Niamh, Rehman Sabah, Climie Rachel E. (2022). Sex differences in pressure and flow waveform physiology across the life course. Journal of Hypertension.

[cit134] Mosca Lori, Benjamin Emelia J., Berra Kathy, Bezanson Judy L., Dolor Rowena J., Lloyd-Jones Donald M., Newby L. Kristin, Piña Ileana L., Roger Véronique L., Shaw Leslee J., Zhao Dong, Beckie Theresa M., Bushnell Cheryl, D'Armiento Jeanine, Kris-Etherton Penny M., Fang Jing, Ganiats Theodore G., Gomes Antoinette S., Gracia Clarisa R., Haan Constance K., Jackson Elizabeth A., Judelson Debra R., Kelepouris Ellie, Lavie Carl J., Moore Anne, Nussmeier Nancy A., Ofili Elizabeth, Oparil Suzanne, Ouyang Pamela, Pinn Vivian W., Sherif Katherine, Smith Sidney C., Sopko George, Chandra-Strobos Nisha, Urbina Elaine M., Vaccarino Viola, Wenger Nanette K. (2011). Effectiveness-Based Guidelines for the Prevention of Cardiovascular Disease in Women—2011 Update. Circulation.

[cit135] Issa Zeinab, Seely Ellen W., Rahme Maya, El-Hajj Fuleihan Ghada (2014). Effects of hormone therapy on blood pressure. Menopause.

[cit136] Mancia Giuseppe, Kreutz Reinhold, Brunström Mattias, Burnier Michel, Grassi Guido, Januszewicz Andrzej, Muiesan Maria Lorenza, Tsioufis Konstantinos, Agabiti-Rosei Enrico, Algharably Engi Abd Elhady, Azizi Michel, Benetos Athanase, Borghi Claudio, Hitij Jana Brguljan, Cifkova Renata, Coca Antonio, Cornelissen Veronique, Cruickshank J. Kennedy, Cunha Pedro G., Danser A.H. Jan, Pinho Rosa Maria de, Delles Christian, Dominiczak Anna F., Dorobantu Maria, Doumas Michalis, Fernández-Alfonso María S., Halimi Jean-Michel, Járai Zoltán, Jelaković Bojan, Jordan Jens, Kuznetsova Tatiana, Laurent Stephane, Lovic Dragan, Lurbe Empar, Mahfoud Felix, Manolis Athanasios, Miglinas Marius, Narkiewicz Krzystof, Niiranen Teemu, Palatini Paolo, Parati Gianfranco, Pathak Atul, Persu Alexandre, Polonia Jorge, Redon Josep, Sarafidis Pantelis, Schmieder Roland, Spronck Bart, Stabouli Stella, Stergiou George, Taddei Stefano, Thomopoulos Costas, Tomaszewski Maciej, Van de Borne Philippe, Wanner Christoph, Weber Thomas, Williams Bryan, Zhang Zhen-Yu, Kjeldsen Sverre E. (2023). 2023 ESH Guidelines for the management of arterial hypertension The Task Force for the management of arterial hypertension of the European Society of Hypertension. Journal of Hypertension.

[cit137] Kobalava Zh. D., Konradi A. O., Nedogoda S. V., Shlyakhto E. V., Arutyunov G. P., Baranova E. I., Barbarash O. L., Boitsov S. A., Vavilova T. V., Villevalde S. V., Galyavich A. S., Glezer M. G., Grineva E. N., Grinstein Yu. I., Drapkina O. M., Zhernakova Yu. V., Zvartau N. E., Kislyak O. A., Koziolova N. A., Kosmacheva E. D., Kotovskaya Yu. V., Libis R. A., Lopatin Yu. M., Nebiridze D. V., Nedoshivin A. O., Ostroumova O. D., Oschepkova E. V., Ratova L. G., Skibitsky V. V., Tkacheva O. N., Chazova I. E., Chesnikova A. I., Chumakova G. A., Shalnova S. A., Shestakova M. V., Yakushin S. S., Yanishevsky S. N. (2020). Arterial hypertension in adults. Clinical guidelines 2020. Russian Journal of Cardiology.

[cit138] Curb J. David, Prentice Ross L., Bray Paul F., Langer Robert D., Van Horn Linda, Barnabei Vanessa M., Bloch Michael J., Cyr Michele G., Gass Margery, Lepine Lisa, Rodabough Rebecca J., Sidney Stephen, Uwaifo Gabriel I., Rosendaal Frits R. (2006). Venous Thrombosis and Conjugated Equine Estrogen in Women Without a Uterus. Archives of Internal Medicine.

[cit139] Blondon Marc, Wiggins Kerri L., Van Hylckama Vlieg Astrid, McKnight Barbara, Psaty Bruce M., Rice Kenneth M., Heckbert Susan R., Smith Nicholas L. (2013). Smoking, postmenopausal hormone therapy and the risk of venous thrombosis: a population‐based, case–control study. British Journal of Haematology.

[cit140] Ruan X., Mueck A. O. (2014). Impact of smoking on estrogenic efficacy. Climacteric.

[cit141] Schreinlechner Michael, Noflatscher Maria, Reinstadler Sebastian Johannes, Sommer Philip, Lener Daniela, Reiser Elisabeth, Theurl Markus, Kirchmair Rudolf, Bauer Axel, Marschang Peter (2020). Early onset of menopause is associated with increased peripheral atherosclerotic plaque volume and progression. Atherosclerosis.

[cit142] Rockman Caron B., Maldonado Thomas S., Jacobowitz Glenn R., Adelman Mark A., Riles Thomas S. (2012). Hormone Replacement Therapy is Associated With a Decreased Prevalence of Peripheral Arterial Disease in Postmenopausal Women. Annals of Vascular Surgery.

[cit143] Westendorp Iris C. D., in't Veld Bas A., Grobbee Diederik E., Pols Huib A. P., Meijer Wonter T., Hofman Albert, Witteman Jacqueline C. M. (2003). Hormone Replacement Therapy and Peripheral Arterial Disease. Archives of Internal Medicine.

[cit144] Hodis Howard N., Mack Wendy J., Henderson Victor W., Shoupe Donna, Budoff Matthew J., Hwang-Levine Juliana, Li Yanjie, Feng Mei, Dustin Laurie, Kono Naoko, Stanczyk Frank Z., Selzer Robert H., Azen Stanley P. (2016). Vascular Effects of Early versus Late Postmenopausal Treatment with Estradiol. New England Journal of Medicine.

[cit145] Chen Irene J, Stanczyk Frank Z, Sriprasert Intira, Karim Roksana, Shoupe Donna, Kono Naoko, Hodis Howard N, Mack Wendy J (2025). Sex steroid hormones and subclinical atherosclerosis progression in postmenopausal women. European Journal of Endocrinology.

[cit146] Anagnostis Panagiotis, Mikhailidis Dimitri P., Blinc Ales, Jensterle Mojca, Ježovnik Mateja K., Schernthaner Gerit-Holger, Antignani Pier Luigi, Studen Katica Bajuk, Šabović Miso, Poredos Pavel (2023). The Effect of Menopause and Menopausal Hormone Therapy on the Risk of Peripheral Artery Disease. Current Vascular Pharmacology.

[cit147] Grady Deborah, Herrington David, Bittner Vera, Blumenthal Roger, Davidson Michael, Hlatky Mark, Hsia Judith, Hulley Stephen, Herd Alan, Khan Steven, Newby L. Kristin, Waters David, Vittinghoff Eric, Wenger Nanette, for the HERS Research Group (2003). Cardiovascular Disease Outcomes During 6.8 Years of Hormone Therapy. JAMA.

[cit148] Davies R.S.M., Vohra R.K., Bradbury A.W., Adam D.J. (2007). The Impact of Hormone Replacement Therapy on the Pathophysiology of Peripheral Arterial Disease. European Journal of Vascular and Endovascular Surgery.

[cit149] Polyakov D. S., Fomin I. V., Belenkov Yu. N., Mareev V. Yu., Ageev F. T., Artemjeva E. G., Badin Yu. V., Bakulina E. V., Vinogradova N. G., Galyavich A. S., Ionova T. S., Kamalov G. M., Kechedzhieva S. G., Koziolova N. A., Malenkova V. Yu., Malchikova S. V., Mareev Yu. V., Smirnova E. A., Tarlovskaya E. I., Shcherbinina E. V., Yakushin S. S. (2021). Chronic heart failure in the Russian Federation: what has changed over 20 years of follow-up? Results of the EPOCH-CHF study. Kardiologiia.

[cit150] Appiah Duke, Schreiner Pamela J., Demerath Ellen W., Loehr Laura R., Chang Patricia P., Folsom Aaron R. (2016). Association of Age at Menopause With Incident Heart Failure: A Prospective Cohort Study and Meta‐Analysis. Journal of the American Heart Association.

[cit151] Schierbeck L. L., Rejnmark L., Tofteng C. L., Stilgren L., Eiken P., Mosekilde L., Kober L., Jensen J.-E. B. (2012). Effect of hormone replacement therapy on cardiovascular events in recently postmenopausal women: randomised trial. BMJ.

[cit152] Lindenfeld JoAnn, Ghali Jalal K, Krause-Steinrauf Heidi J, Khan Steven, Adams Kirkwood, Goldman Steven, Peberdy Mary Ann, Yancy Clyde, Thaneemit-Chen Surai, Larsen Rhonda L, Young James, Lowes Brian, Rosenberg Yves D (2003). Hormone replacement therapy is associated with improved survival in women with advanced heart failure. Journal of the American College of Cardiology.

[cit153] Liu Longjian, Klein Liviu, Eaton Charles, Panjrath Gurusher, Martin Lisa Warsinger, Chae Claudia U., Greenland Philip, Lloyd-Jones Donald M, Wactawski-Wende Jean, Manson JoAnn E. (2019). Menopausal Hormone Therapy and Risks of First Hospitalized Heart Failure and its Subtypes During the Intervention and Extended Postintervention Follow-up of the Women's Health Initiative Randomized Trials. Journal of Cardiac Failure.

[cit154] Baik Seo H., Baye Fitsum, McDonald Clement J. (2024). Use of menopausal hormone therapy beyond age 65 years and its effects on women's health outcomes by types, routes, and doses. Menopause.

[cit155] Kirchhof Paulus, Benussi Stefano, Kotecha Dipak, Ahlsson Anders, Atar Dan, Casadei Barbara, Castella Manuel, Diener Hans-Christoph, Heidbuchel Hein, Hendriks Jeroen, Hindricks Gerhard, Manolis Antonis S., Oldgren Jonas, Popescu Bogdan Alexandru, Schotten Ulrich, Van Putte Bart, Vardas Panagiotis, Agewall Stefan, Camm John, Baron Esquivias Gonzalo, Budts Werner, Carerj Scipione, Casselman Filip, Coca Antonio, De Caterina Raffaele, Deftereos Spiridon, Dobrev Dobromir, Ferro José M., Filippatos Gerasimos, Fitzsimons Donna, Gorenek Bulent, Guenoun Maxine, Hohnloser Stefan H., Kolh Philippe, Lip Gregory Y. H., Manolis Athanasios, McMurray John, Ponikowski Piotr, Rosenhek Raphael, Ruschitzka Frank, Savelieva Irina, Sharma Sanjay, Suwalski Piotr, Tamargo Juan Luis, Taylor Clare J., Van Gelder Isabelle C., Voors Adriaan A., Windecker Stephan, Zamorano Jose Luis, Zeppenfeld Katja (2016). 2016 ESC Guidelines for the management of atrial fibrillation developed in collaboration with EACTS. European Heart Journal.

[cit156] Fang Margaret C., Singer Daniel E., Chang Yuchiao, Hylek Elaine M., Henault Lori E., Jensvold Nancy G., Go Alan S. (2005). Gender Differences in the Risk of Ischemic Stroke and Peripheral Embolism in Atrial Fibrillation. Circulation.

[cit157] Van Gelder Isabelle C, Rienstra Michiel, Bunting Karina V, Casado-Arroyo Ruben, Caso Valeria, Crijns Harry J G M, De Potter Tom J R, Dwight Jeremy, Guasti Luigina, Hanke Thorsten, Jaarsma Tiny, Lettino Maddalena, Løchen Maja-Lisa, Lumbers R Thomas, Maesen Bart, Mølgaard Inge, Rosano Giuseppe M C, Sanders Prashanthan, Schnabel Renate B, Suwalski Piotr, Svennberg Emma, Tamargo Juan, Tica Otilia, Traykov Vassil, Tzeis Stylianos, Kotecha Dipak, Dagres Nikolaos, Rocca Bianca, Ahsan Syed, Ameri Pietro, Arbelo Elena, Bauer Axel, Borger Michael A, Buccheri Sergio, Casadei Barbara, Chioncel Ovidiu, Dobrev Dobromir, Fauchier Laurent, Gigante Bruna, Glikson Michael, Hijazi Ziad, Hindricks Gerhard, Husser Daniela, Ibanez Borja, James Stefan, Kaab Stefan, Kirchhof Paulus, Køber Lars, Koskinas Konstantinos C, Kumler Thomas, Lip Gregory Y H, Mandrola John, Marx Nikolaus, Mcevoy John William, Mihaylova Borislava, Mindham Richard, Muraru Denisa, Neubeck Lis, Nielsen Jens Cosedis, Oldgren Jonas, Paciaroni Maurizio, Pasquet Agnes A, Prescott Eva, Rega Filip, Rossello Francisco Javier, Rucinski Marcin, Salzberg Sacha P, Schulman Sam, Sommer Philipp, Svendsen Jesper Hastrup, ten Berg Jurrien M, Ten Cate Hugo, Vaartjes Ilonca, Vrints Christiaan Jm, Witkowski Adam, Zeppenfeld Katja, Simoni Leonard, Kichou Brahim, Sisakian Hamayak S, Scherr Daniel, Cools Frank, Smajić Elnur, Shalganov Tchavdar, Manola Sime, Avraamides Panayiotis, Taborsky Milos, Brandes Axel, El-Damaty Ahmed M, Kampus Priit, Raatikainen Pekka, Garcia Rodrigue, Etsadashvili Kakhaber, Eckardt Lars, Kallergis Eleftherios, Gellér László, Guðmundsson Kristján, Lyne Jonathan, Marai Ibrahim, Colivicchi Furio, Abdrakhmanov Ayan Suleimenovich, Bytyci Ibadete, Kerimkulova Alina, Kupics Kaspars, Refaat Marwan, Bheleel Osama Abdulmajed, Barysienė Jūratė, Leitz Patrick, Sammut Mark A, Grosu Aurel, Pavlovic Nikola, Moustaghfir Abdelhamid, Yap Sing-Chien, Taleski Jane, Fink Trine, Kazmierczak Jaroslaw, Sanfins Victor M, Cozma Dragos, Zavatta Marco, Kovačević Dragan V, Hlivak Peter, Zupan Igor, Calvo David, Björkenheim Anna, Kühne Michael, Ouali Sana, Demircan Sabri, Sychov Oleg S, Ng Andre, Kuchkarov Husniddin (2024). 2024 ESC Guidelines for the management of atrial fibrillation developed in collaboration with the European Association for Cardio-Thoracic Surgery (EACTS). European Heart Journal.

[cit158] Antoun Ibrahim, Layton Georgia R., Abdelrazik Ahmed, Eldesouky Mahmoud, Zakkar Mustafa, Somani Riyaz, Ng André (2025). The Pathophysiology of Sex Differences in Stroke Risk and Prevention in Atrial Fibrillation: A Comprehensive Review. Medicina.

[cit159] Perez Marco V., Wang Paul J., Larson Joseph C., Virnig Beth A., Cochrane Barbara, Curb J. David, Klein Liviu, Manson JoAnn E., Martin Lisa W., Robinson Jennifer, Wassertheil-Smoller Sylvia, Stefanick Marcia L. (2012). Effects of Postmenopausal Hormone Therapy on Incident Atrial Fibrillation. Circulation: Arrhythmia and Electrophysiology.

[cit160] Magnussen Christina, Niiranen Teemu J., Ojeda Francisco M., Gianfagna Francesco, Blankenberg Stefan, Njølstad Inger, Vartiainen Erkki, Sans Susana, Pasterkamp Gerard, Hughes Maria, Costanzo Simona, Donati Maria Benedetta, Jousilahti Pekka, Linneberg Allan, Palosaari Tarja, de Gaetano Giovanni, Bobak Martin, den Ruijter Hester M., Mathiesen Ellisiv, Jørgensen Torben, Söderberg Stefan, Kuulasmaa Kari, Zeller Tanja, Iacoviello Licia, Salomaa Veikko, Schnabel Renate B. (2017). Sex Differences and Similarities in Atrial Fibrillation Epidemiology, Risk Factors, and Mortality in Community Cohorts. Circulation.

[cit161] Tsai Wei-Chung, Haung Yaw-Bin, Kuo Hsuan-Fu, Tang Wei-Hua, Hsu Po-Chao, Su Ho-Ming, Lin Tsung-Hsien, Chu Chih-Sheng, Jhuo Shih-Jie, Lee Kun-Tai, Sheu Sheng-Hsiung, Chen Chung-Yu, Wu Ming-Tsang, Lai Wen-Ter (2016). Hormone replacement therapy and risk of atrial fibrillation in Taiwanese menopause women: A nationwide cohort study. Scientific Reports.

[cit162] Wong Jorge A, Rexrode Kathryn M, Sandhu Roopinder K, Moorthy M Vinayaga, Conen David, Albert Christine M (2017). Menopausal age, postmenopausal hormone therapy and incident atrial fibrillation. Heart.

[cit163] Lee Jaehoon, Kim Yuntae, Park Hyunji, Kim Changsoo, Cho Sihyun, Kim Jongyoun (2021). Clinical Impact of Hormone Replacement Therapy on Atrial Fibrillation in Postmenopausal Women: A Nationwide Cohort Study. Journal of Clinical Medicine.

[cit164] Fleury Marie-Ange, Clavel Marie-Annick (2021). Sex and Race Differences in the Pathophysiology, Diagnosis, Treatment, and Outcomes of Valvular Heart Diseases. Canadian Journal of Cardiology.

[cit165] Rodriguez-Arias Juan José, García-Álvarez Ana (2021). Sex Differences in Pulmonary Hypertension. Frontiers in Aging.

[cit166] Hye Tanvirul, Dwivedi Pankaj, Li Wei, Lahm Tim, Nozik-Grayck Eva, Stenmark Kurt R., Ahsan Fakhrul (2021). Newer insights into the pathobiological and pharmacological basis of the sex disparity in patients with pulmonary arterial hypertension. American Journal of Physiology-Lung Cellular and Molecular Physiology.

[cit167] Liu Aiping, Schreier David, Tian Lian, Eickhoff Jens C., Wang Zhijie, Hacker Timothy A., Chesler Naomi C. (2014). Direct and indirect protection of right ventricular function by estrogen in an experimental model of pulmonary arterial hypertension. American Journal of Physiology-Heart and Circulatory Physiology.

[cit168] Badlam Jessica B., Badesch David, Brittain Evan, Cordell Shannon, Ding Tan, Fox Kelly, Hemnes Anna, Loyd James, Pugh Meredith, Robbins Ivan, Yu Chang, Austin Eric D. (2020). Sex hormone exposure and reproductive factors in pulmonary arterial hypertension: a case–control study. Pulmonary Circulation.

[cit169] Ministerstvo zdravookhraneniya Rossiiskoi Federatsii. Klinicheskie rekomendatsii. Migren'. 2024. Dostupno na: https://cr.minzdrav.gov.ru/preview-cr/295_4

[cit170] Nappi Rossella, Tiranini Lara, Sacco Simona, De Matteis Eleonora, De Icco Roberto, Tassorelli Cristina (2022). Role of Estrogens in Menstrual Migraine. Cells.

[cit171] Lee Sa Ra, Cho Moon Kyoung, Cho Yeon Jean, Chun Sungwook, Hong Seung-Hwa, Hwang Kyu Ri, Jeon Gyun-Ho, Joo Jong Kil, Kim Seul Ki, Lee Dong Ock, Lee Dong-Yun, Lee Eun Sil, Song Jae Yen, Yi Kyong Wook, Yun Bo Hyon, Shin Jung-Ho, Chae Hee Dong, Kim Tak (2020). The 2020 Menopausal Hormone Therapy Guidelines. Journal of Menopausal Medicine.

[cit172] Australasian Menopause Society. Migraine headaches, menopause and MHT/HRT. Available at: https://menopause.org.au/hp/information-sheets/migraine-headaches-menopause-and-mht-hrt

[cit173] British Menopause Society. Migraine and HRT. Av. at: https://thebms.org.uk/publications/tools-for-clinicians/migraine-and-hrt/

[cit174] Garvey W. Timothy, Garber Alan J., Mechanick Jeffrey I., Bray George A., Dagogo-Jack Samuel, Einhorn Daniel, Grunberger George, Handelsman Yehuda, Hennekens Charles H., Hurley Daniel L., McGill Janet, Palumbo Pasquale, Umpierrez Guillermo (2014). American Association of Clinical Endocrinologists and American College of Endocrinology Position Statement on the 2014 Advanced Framework for a New Diagnosis of Obesity as a Chronic Disease. Endocrine Practice.

